# Polarization-Sensitive OCT Imaging of Scleral Abnormalities in Eyes With High Myopia and Dome-Shaped Macula

**DOI:** 10.1001/jamaophthalmol.2024.0002

**Published:** 2024-03-07

**Authors:** Kyoko Ohno-Matsui, Tae Igarashi-Yokoi, Takeshi Azuma, Keigo Sugisawa, Jianping Xiong, Tomonari Takahashi, Kengo Uramoto, Koju Kamoi, Michiaki Okamoto, Suchandra Banerjee, Masahiro Yamanari

**Affiliations:** 1 Department of Ophthalmology and Visual Science, Tokyo Medical and Dental University, Tokyo, Japan; 2 Tomey Corporation, Nagoya, Aichi-ken, Japan

## Abstract

**Importance:**

The relevance of visualizing scleral fiber orientation may offer insights into the pathogenesis of pathologic myopia, including dome-shaped maculopathy (DSM).

**Objective:**

To investigate the orientation and density of scleral collagen fibers in highly myopic eyes with and without DSM by polarization-sensitive optical coherence tomography (PS-OCT).

**Design, Setting, and Participants:**

This case series included patients with highly myopic eyes (defined as a refractive error ≥6 diopters or an axial length ≥26.5 mm) with and without a DSM examined at a single site in May and June 2019. Analysis was performed from September 2019 to October 2023.

**Exposures:**

The PS-OCT was used to study the birefringence and optic axis of the scleral collagen fibers.

**Main Outcomes and Measures:**

The orientation and optic axis of scleral fibers in inner and outer layers of highly myopic eyes were assessed, and the results were compared between eyes with and without a DSM.

**Results:**

A total of 72 patients (51 [70.8%] female; mean [SD] age, 61.5 [12.8] years) were included, and 89 highly myopic eyes were examined (mean [SD] axial length, 30.4 [1.7] mm); 52 (58.4%) did not have a DSM and 37 (41.6%) had a DSM (10 bidirectional [27.0%] and 27 horizontal [73.0%]). Among the 52 eyes without DSM, the 13 eyes with simple high myopia had primarily inner sclera visible, displaying radially oriented fibers in optic axis images. In contrast, the entire thickness of the sclera was visible in 39 eyes with pathologic myopia. In these eyes, the optic axis images showed vertically oriented fibers within the outer sclera. Eyes presenting with both horizontal and bidirectional DSMs had clusters of fibers with low birefringence at the site of the DSM. In the optic axis images, horizontally or obliquely oriented scleral fibers were aggregated in the inner layer at the DSM. The vertical fibers located posterior to the inner fiber aggregation were not thickened and appeared thin compared with the surrounding areas.

**Conclusions and Relevance:**

This study using PS-OCT revealed inner scleral fiber aggregation without outer scleral thickening at the site of the DSM in highly myopic eyes. Given the common occurrence of scleral pathologies, such as DSM, and staphylomas in eyes with pathologic myopia, recognizing these fiber patterns could be important. These insights may be relevant to developing targeted therapies to address scleral abnormalities early and, thus, mitigate potential damage to the overlying neural tissue.

## Key Points

**Question:**

What does polarization-sensitive optical coherence tomography (PS-OCT) show in highly myopic eyes with scleral pathologies?

**Findings:**

In this case series of 89 highly myopic eyes of 72 patients, PS-OCT of eyes without dome-shaped macula showed low-density, radially oriented fibers in the inner layer and high-density, vertically oriented fibers in the outer layer of the sclera. Scleral fibers in the inner layer were aggregated and thickened in eyes with dome-shaped macula, while fibers in the outer sclera were compressed without thickening.

**Meaning:**

The findings suggest that PS-OCT may advance understanding of scleral pathologies in eyes with pathologic myopia.

## Teaser

This case series examines the orientation and density of scleral collagen fibers found on polarization-sensitive optical coherence tomography (PS-OCT) of highly myopic eyes with and without dome-shaped macula.

## Introduction

The sclera is a major component of the eye’s outer coat and consists mainly of collagen. The scleral stroma is composed of collagen bundles and fibroblasts along with a moderate amount of ground substance. The dimensions and course of the scleral collagen bundles have not been fully determined, but histologic studies have shown that the superficial and deep layers of the sclera are different.^1^

Earlier studies of scleral morphologic features were primarily based on histologic observations. However, with the advancement of optical coherence tomography (OCT), it is possible to obtain images and study the morphologic features of the inner part of the sclera. In highly myopic eyes with a thin choroid and sclera, the entire thickness of the sclera, including the outer sclera, is visible in many cases. Nevertheless, most studies focus on the thickness of the sclera because observing the details of the collagen fibers in the scleral stroma is still difficult.

A dome-shaped macula (DSM) has been identified as an inward protrusion of the macula that can be detected in OCT images.^3^ Imamura et al^5^ reported that a DSM in highly myopic eyes may result from a thickening of the macular sclera and is potentially associated with a local, less pronounced thinning of the subfoveal sclera than in eyes without a DSM. Although the pathogenesis of DSMs has not been definitively determined, Ohno-Matsui et al^6^ reported a peridome choroidal deepening and Fang et al^7^ reported defects of the Bruch membrane (BM) around a DSM. These findings suggest that the DSM may be associated with a focal relaxation of the posterior sclera and insufficient internal pressure by an expanding BM causing a partial inward bulge of the sclera, leading to the formation of a DSM.

Polarization-sensitive OCT (PS-OCT) is a functionally extended OCT that uses the polarization of light as the image contrast mechanism in addition to the contrast mechanism of conventional OCT.^8^ Although the typical resolution of PS-OCT images does not allow the detection of individual collagen lamellae in the sclera,^10^ the polarized properties of the sclera have been investigated on several tens of micrometer scale in both animal and human studies.^11^ Notably, the sclera has a polarization property called birefringence, which is an optical property of a material in which the refractive index depends on the state of polarization and is present in fibrous tissues that have periodically organized nanostructures. In addition to the magnitude of the birefringence that reflects the density of fibers, PS-OCT can also show the axis of orientation or the optic axis of the birefringence that is related to the orientation of the fiber bundles. Optic axis imaging of the sclera of the fundus has been reported in the eyes of rats,^16^ healthy humans,^17^ guinea pigs, and normal and highly myopic eyes of humans.^18^ However, to our knowledge, there has not been a study focused on a DSM that has specific variations in its morphologic features.

Thus, the purpose of this study was to evaluate the density and orientation of the collagen fibers in the inner and outer layers of the sclera at the site of a DSM. For reference control, highly myopic eyes without a DSM were also studied because the entire scleral thickness is clearly visible in highly myopic eyes.

## Methods

The study protocol for this case series adhered to the tenets of the Declaration of Helsinki,^19^ and it was approved by the ethics committee of the Tokyo Medical and Dental University. We followed the reporting guideline for case series to ensure comprehensive and transparent reporting of our findings. Written informed consent was obtained from all participants. Participants were provided with detailed information about the study objectives, procedures, and potential risks. No compensation or incentives were provided for their participation.

The medical records of patients with highly myopic eyes who had undergone PS-OCT examinations in May and June 2019 were retrospectively analyzed from September 2019 to October 2023. High myopia was defined as a myopic refractive error of greater than 6 diopters (D) or an axial length (AL) of 26.5 mm or more. Pathologic myopia was defined as myopic eyes having myopic maculopathy equal to or more severe than diffuse choroidal atrophy or eyes having staphylomas, according to the Meta-Analysis for Pathologic Myopia (META-PM) study and the International Myopia Institute.^20^

The exclusion criteria included poor-quality OCT images of the posterior sclera due to media opacities, such as dense cataracts. Patients with previous vitreoretinal surgery were also excluded because it might have altered the scleral curvature. Presence of retinal complications, like myopic retinoschisis, was not an exclusion criterion because these complications did not affect the imaging of sclera.

For the measurement of the birefringent properties of the posterior eye, we constructed a prototype PS-OCT system using a swept laser at a 1050-nm center wavelength. The details of this system have been described elsewhere.^24^ The size of the retinal scans was 9 × 9 mm^2^ or 12 × 12 mm^2^ using a raster scanning protocol of 1024 × 256 A-scans (horizontal × vertical directions). The axial resolution was 7.3 μm, and the depth measurement range was 4.49 mm in tissue.

All patients underwent comprehensive ophthalmologic examinations, including measurements of refractive error, measurement of AL (IOL Master 700; Carl Zeiss Meditec Co), color fundus photography, and swept-source OCT (Triton; Topcon). As in previous studies, a DSM was defined as an inward bulging of the retinal pigment epithelium line in the OCT images with a minimal height of 50 μm above a line connecting the retinal pigment epithelium lines on both sides of the DSM.^25^ The DSMs were classified as horizontal, vertical, or bidirectional types according to a modification of the classification presented by Caillaux et al^25^ and Xu et al.^27^ The height and width of the DSM were measured on the OCT images showing the maximal height or width of the DSM among the 12 radial OCT sections centered on the fovea.

The measured data were processed to obtain the polarization-diverse OCT intensity,^28^ the local retardation (magnitude of the birefringence), and the optic axis by algorithms developed previously.^30^ To improve the quality of the images, we increased the axial pixel separation to localize the axially accumulated Jones matrix by implementing a necessary correction to resolve the optic axis at each axial depth. The algorithms used and a supplemental description of birefringence are detailed in eMethods 1 and 2 in Supplement 1, respectively.

Because the optic axis is a vectorial contrast that shows the orientation of the fibrous tissues, plotting or rendering methods dedicated to the vectorial data are preferred. Streamline rendering is 1 of such methods, and it has been used for the imaging of the optic axis of the heart muscle and articular cartilage.^31^ We used ParaView, version 5.11.1 (Kitware Inc)^34^ for volumetric streamline rendering of the optic axis. The volumetric data points were randomly downsampled and input to a stream tracer function as custom seeds together with the raw volumetric data. This was done for the estimation of the vectorial connections by solving the numerical integration using the Runge-Kutta method. The resultant streamline data were volumetrically rendered by the ParaView algorithm.

## Results

**Figure 1.**
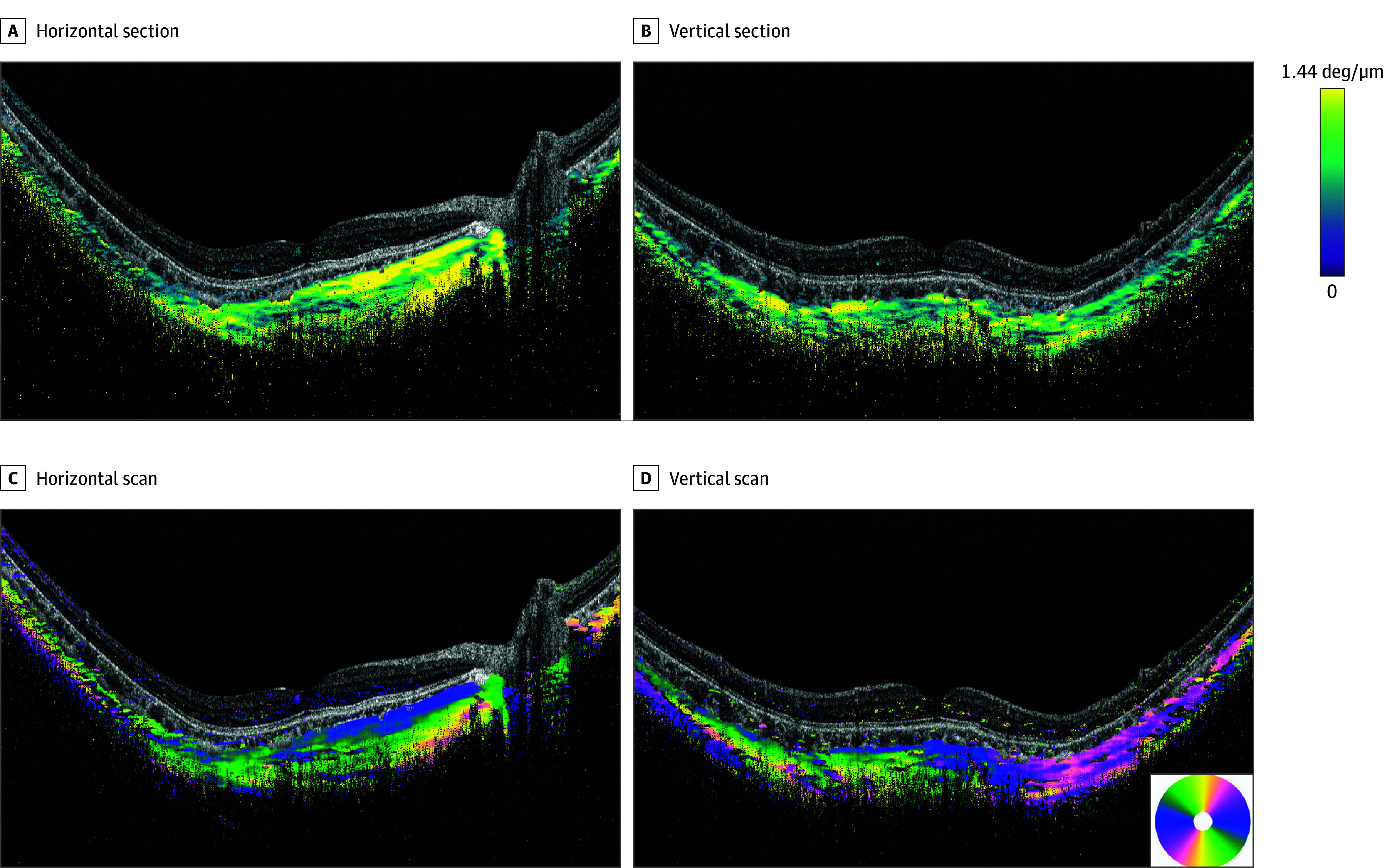
Polarization-Sensitive Optical Coherence Tomography (PS-OCT) Images of a Highly Myopic Eye Without a Dome-Shaped Macula or Pathologic Myopia Images of the right eye of a 30-year-old man with a refractive error of −14.9 diopters and an axial length of 28.9 mm showing a tessellated fundus without myopic atrophic lesions. A and B, Birefringence images obtained by PS-OCT. Images of the horizontal (A) and vertical (B) sections across the fovea showing a mixture of low-density (green) and high-density (yellow) birefringent fibers. The color map is intentionally saturated for better views of low birefringence structures. C and D, PS-OCT optic axis images. Yellow, blue, green, and pink indicate the vertical, horizontal, and the 2 oblique scan directions, respectively. C, In the horizontal scan, a horizontal fiber (blue) can be seen to run from the optic disc toward the macula along the inner surface of the sclera. Obliquely running fibers (green) can be seen especially in the macular area posterior to the horizontal fibers. Vertically running fibers (yellow) are seen only temporal to the optic nerve. D, In a vertical scan image, the horizontal fibers (blue) are seen in the macular region and oblique fibers (pink and green) are seen superior and inferior to the macula. The outer sclera is not clearly seen. deg indicates degrees.

**Figure 2.**
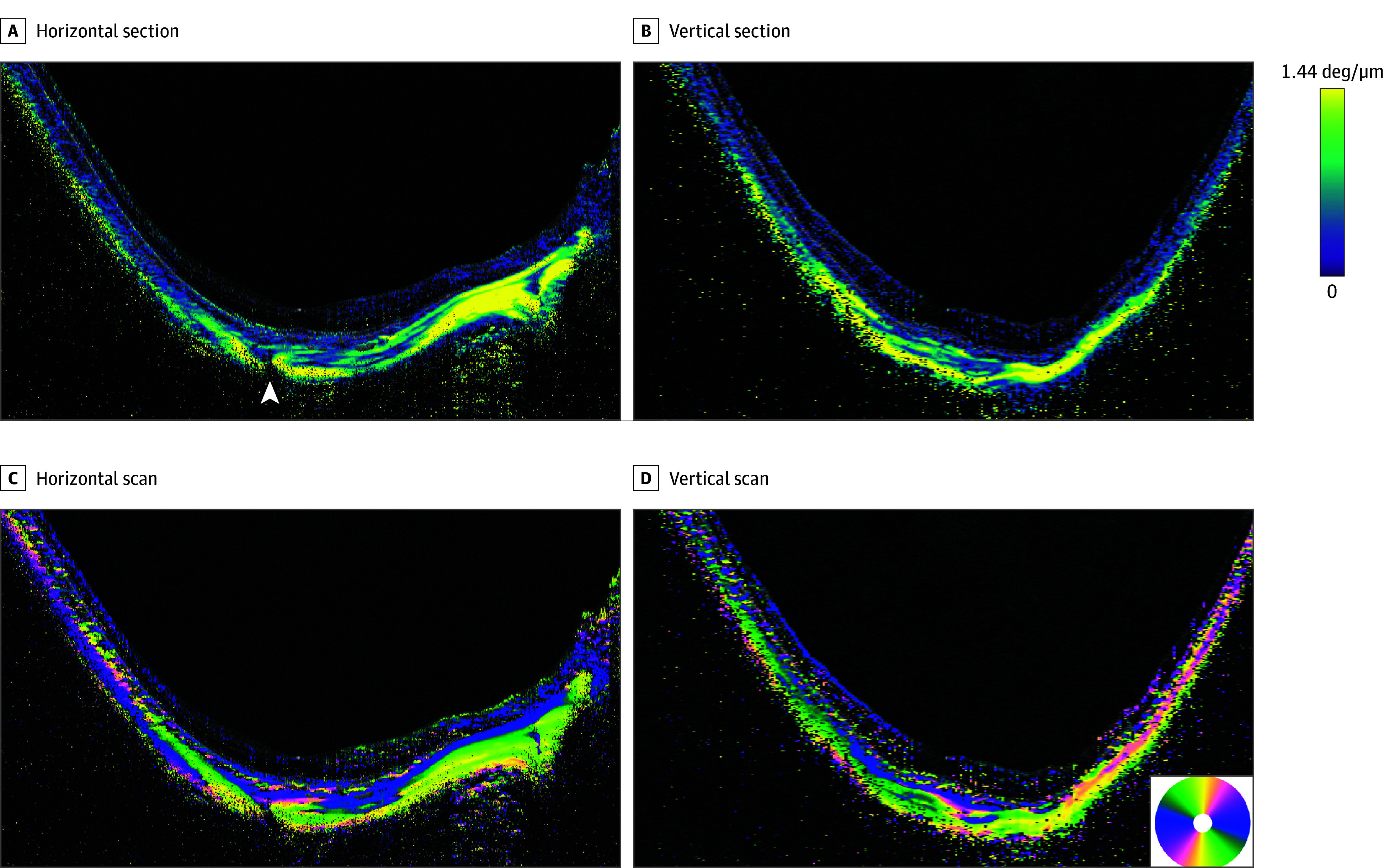
Polarization-Sensitive Optical Coherence Tomography Images of an Eye With Pathologic Myopia and Without a Dome-Shaped Macula Images of the right eye of a 46-year-old woman with a refractive error of −23.5 diopters and an axial length of 32.0 mm showing diffuse choroidal atrophy. A, Horizontal section of birefringence image across the fovea shows low birefringence (green) in general, but high birefringence (yellow) is seen in the outer scleral layer in the macular area and in the area temporal to the optic nerve. The blood vessel emissary (arrowhead) penetrates the sclera slightly temporal to the fovea and is discontinuous with the birefringence. B, Vertical section across the fovea showing that the fibers have low birefringence (green) in the inner layer of the sclera in the macular region. Fibers with high birefringence (yellow) are seen in the outer scleral layer in the macular area and in the outer sclera superior and inferior to the macula. C, Horizontally scanned optic axis image shows horizontal fibers (blue) running from the optic nerve toward the macula along the inner surface of the sclera. Vertically running fibers (yellow) are seen posterior to the horizontal fibers in a wide area of the posterior sclera. D, Vertically scanned image showing horizontal fibers (blue) in the inner layer of the sclera in the macular region. The sclera consists mainly of vertical (yellow) and oblique (green) fibers. deg indicates degrees.

**Figure 3.**
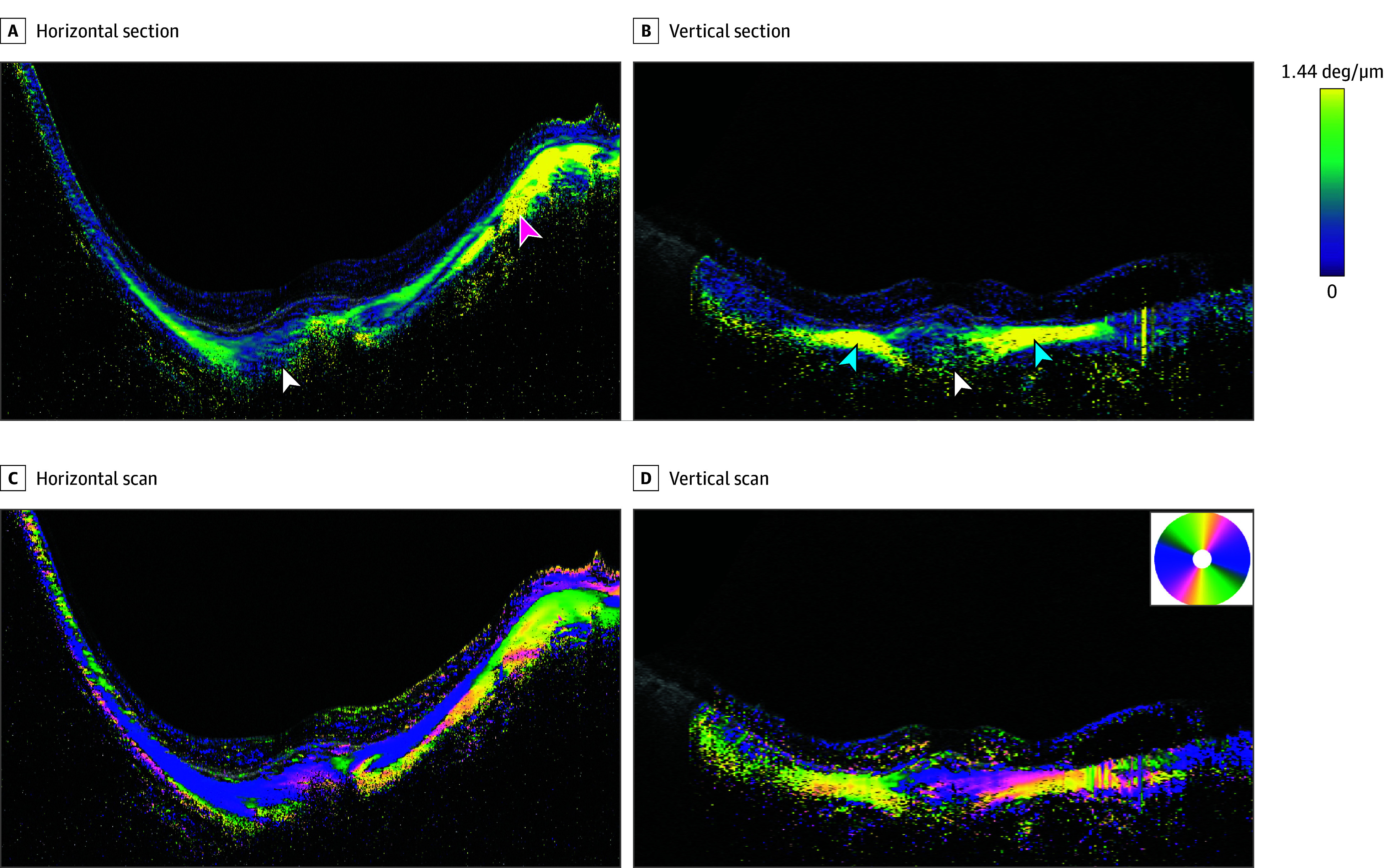
Polarization-Sensitive Optical Coherence Tomography Images of a Highly Myopic Right Eye With a Bidirectional Dome-Shaped Macula (DSM) Images of the right eye of a 76-year-old woman with an implanted intraocular lens and an axial length of 30.1 mm showing diffuse choroidal atrophy. A, The image of a horizontal section across the fovea shows low birefringence fibers (green, indicated by white arrowhead) in general, while high birefringence fibers (yellow, indicated by magenta arrowhead) are seen only temporal to the optic nerve. B, The vertical section across the fovea shows low birefringence fibers (green, indicated by white arrowhead) at the DSM. In contrast, the fibers with high birefringence (yellow, indicated by blue arrowheads) are seen superior and inferior to the DSM. C, In the horizontal scan image of the optic axis, the DSM shows a mixture of horizontal (blue) and oblique (pink) fibers. The horizontal fibers are seen extensively nasal and temporal to the DSM along the inner sclera. The vertical fibers (yellow) can be seen in the outer sclera, especially temporal to the optic nerve. D, In a vertically scanned image, a mixture of horizontal (blue) and oblique (green and pink) fibers is seen at the DSM. The outer layer of the sclera superior and inferior to the DSM consists of vertical fibers (yellow). deg indicates degrees.

**Figure 4.**
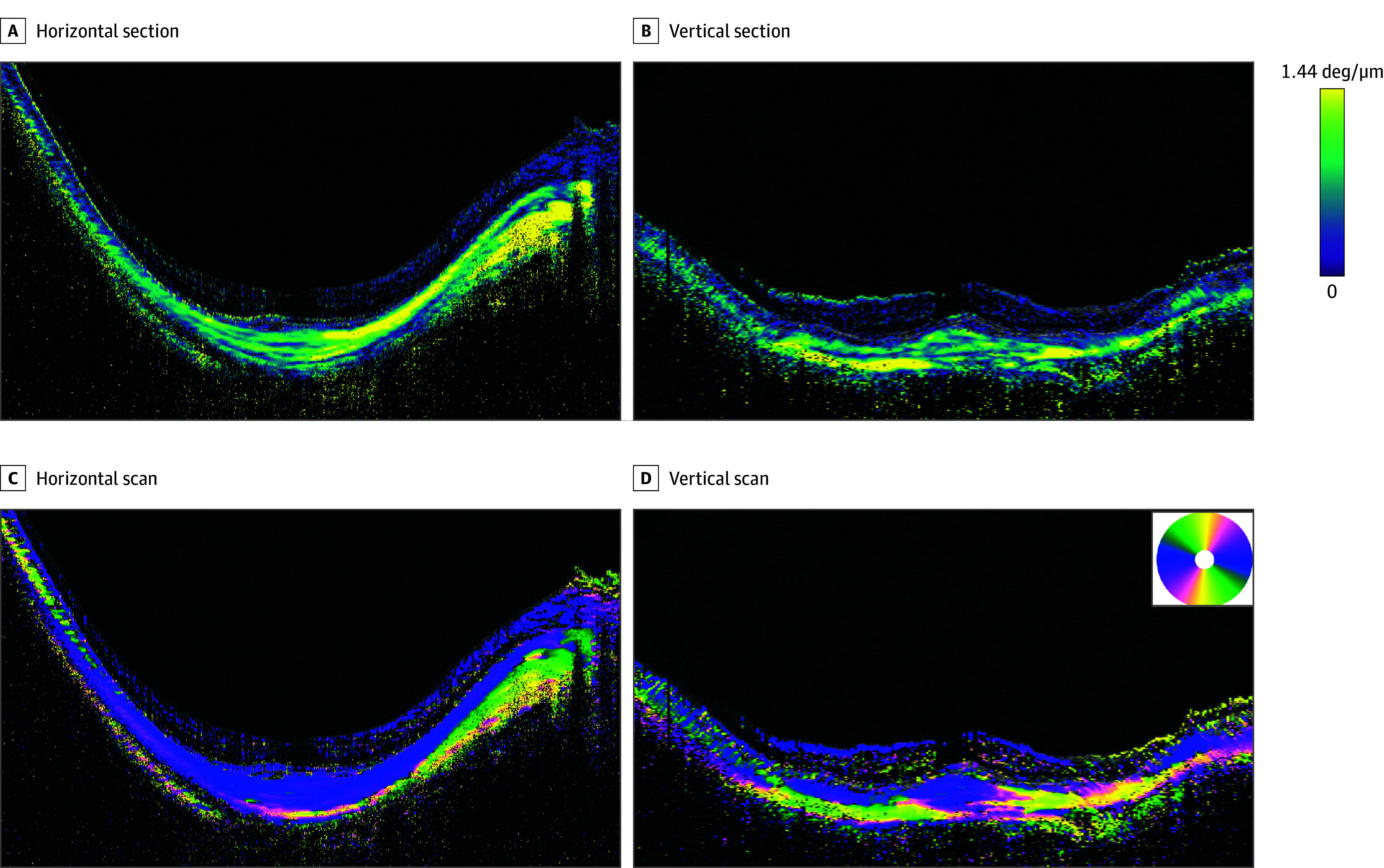
Polarization-Sensitive Optical Coherence Tomography Images of a Highly Myopic Right Eye With a Horizontal Dome-Shaped Macula (DSM) Images of the right eye of a 48-year-old woman with an implanted intraocular lens and an axial length of 30.8 mm showing diffuse choroidal atrophy. A, The horizontal section of the birefringence image across the fovea shows mainly low birefringence fibers (green) in the macula. Fibers with high birefringence are seen between the optic nerve and the macula. B, The vertical section across the fovea shows an aggregation and thickening of fibers with low birefringence (green) at the DSM. The sclera superior and inferior to the DSM has high birefringence (yellow). C, In the horizontal scan image of the optic axis, the relatively thick horizontal fibers (blue) are seen in a wide area. A thin layer of vertical fibers (yellow) is seen in the outer sclera of the macula. Vertical fibers are seen in the outer sclera between the optic nerve and the macula. D, In a vertically scanned image, the DSM appears as an aggregation and thickening of horizontal (blue) and oblique (pink) fibers. Posterior to these fibers, vertical fibers (yellow) are seen in the outer sclera. The outer sclera consists of vertical fibers that are not thickened but appear compressed. The outer sclera superior and inferior to the DSM consists mainly of vertical fibers (yellow). deg indicates degrees.

A total of 89 eyes of 72 patients with high myopia met the inclusion criteria and were studied; 51 (70.8%) were female, and 21 (29.2%) were male. The mean (SD) age of the patients was 61.5 (12.8) years, and the mean (SD) refractive error of 37 eyes (not including 35 pseudophakic eyes) was −13.3 (5.2) D. The mean (SD) AL was 30.4 (1.7) mm. Clinical and ocular characteristics of the subgroups are presented in eTable 1 in Supplement 1. A total of 52 eyes (58.4%) did not have a DSM and 37 (41.6%) had a DSM. Thirteen eyes (14.6%) of 8 patients had simple high myopia without pathologic myopia or a DSM (Figure 1), 39 eyes (43.8%) of 31 patients had pathologic myopia without a DSM (Figure 2), 27 eyes (30.3%) of 25 patients had a horizontal DSM (Figure 3), and 10 eyes (11.2%) of 8 patients had a bidirectional DSM (Figure 4). The clinical and ocular characteristics of these groups are presented in eTable 1 in Supplement 1.

### Scleral Morphologic Features of Eyes With Simple High Myopia Without Pathologic Myopia

Thirteen eyes of 8 patients had high myopia without any signs of pathologic myopia according to META-PM classification.^20^ The mean (SD) age of the patients was 51.6 (16.0) years, mean (SD) refractive error was −10.9 (3.0) D, and the mean (SD) AL was 28.5 (1.2 mm). In all 13 eyes, the inner part of the sclera was visible, but the outer scleral surface was visible in only 3 eyes (23.1%) in the B-scan OCT images (Figure 1 and eFigure 1 in Supplement 1). Thus, the level of birefringence and the optic axis of the inner sclera were mainly analyzed in the 13 eyes.

The birefringent images of the inner sclera of 6 of the 13 eyes (46.2%) had a mixture of low and high birefringence in both the horizontal and vertical scan images (Figure 1A and B). Five eyes (38.5%) had low birefringence in the scan images in both directions. The remaining 2 eyes (15.4%) had mixed patterns, with one eye showing a mixture in the horizontal scan images and the other in the vertical scan images. Overall, the birefringent images of the inner sclera of eyes with simple high myopia typically indicated low birefringence or a combination of low and high birefringence.

**Figure 5.**
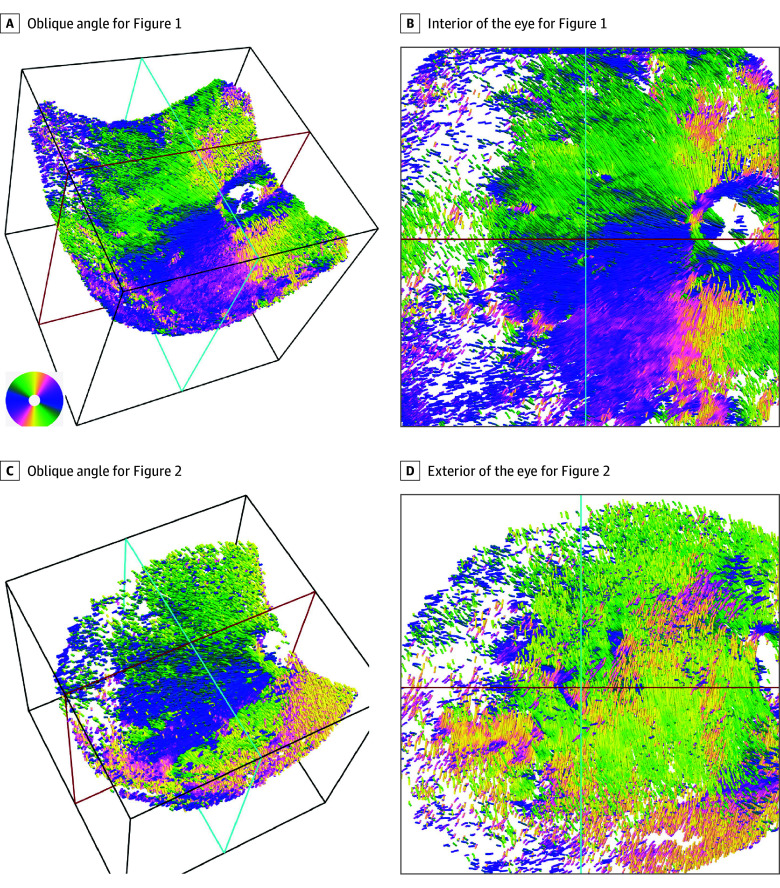
Streamline Renderings of the Optic Axis for the Eyes Shown in Figures 1 and 2 A and B, Streamlined images recorded with an oblique angle of the camera (A) and from the interior of the eye (B) rendered by ParaView for the participant shown in Figure 1. B, The course of the inner scleral fibers is seen clearly. A concentric peripapillary ring can be seen around the optic nerve head. The inner scleral fibers spread radially from this ring toward the periphery. C and D, Streamlined images recorded with an oblique angle of the camera (C) and from the exterior of the eye (D) rendered by ParaView for the participant shown in Figure 2. The course of the outer scleral fibers can be clearly seen to run almost vertically in parallel with the concentric peripapillary circles.

The horizontally scanned optic axis images of the inner sclera showed that the fibers ran horizontally from the optic nerve to the macula and beyond within the innermost layer of the sclera (Figure 1C). Macular scans revealed a mixture of horizontal and oblique fibers throughout the inner scleral layer in all 13 eyes. The vertically scanned images showed horizontal fibers at the macula and oblique fibers above and below it (Figure 1D). Overall, the inner scleral optic axis images in eyes with uncomplicated high myopia showed fibers with a radial orientation from the optic nerve toward the periphery (Figure 5A and B).

### Eyes With Pathologic Myopia Without DSM

Thirty-nine eyes of 31 patients with pathologic myopia without a DSM were studied. The mean (SD) age of the patients was 63.7 (11.2) years, the mean (SD) refractive error of 25 eyes (14 eyes were pseudophakic) was −13.6 (4.4) D, and the mean (SD) AL was 30.4 (1.5) mm. The outer scleral surface was clearly seen in all 39 eyes, and the mean (SD) subfoveal scleral thickness was 256.0 (68.1) μm.

In our analysis of eyes with pathologic myopia, PS-OCT focused on examining the outer sclera, which is uniquely visible in this subgroup. Birefringent imaging of the outer sclera unveiled distinct patterns. Among the 39 eyes with visible outer sclera, 22 (56.4%) displayed high birefringence, noticeable in both horizontal and vertical scans. Notably, in the remaining 13 eyes (33.3%), the outer sclera within the macular region showcased a combination of high and low birefringence, with only 4 eyes (30.8%) exclusively exhibiting low birefringence.

The PS-OCT optic axis images of macular sclera of 30 of these 39 eyes (76.9%) showed that the horizontal and oblique fibers in the inner scleral layer were thinner than those in the eyes with simple high myopia (Figure 2C and D and eFigure 2). In the outer scleral layer in the macular region, 32 of the 39 eyes (82.1%) had vertical fibers in both the horizontal and the vertical scanned images and 3 eyes (7.7%) had vertical fibers only in the vertical scanned images. Overall, 35 eyes (89.7%) had vertical fibers in the outer scleral layer in the image of at least 1 scan direction, while the remaining 4 eyes (10.3%) had a mixture of vertical and oblique fibers.

We also examined the scleral findings in areas surrounding the macula. Among the 39 eyes, 33 (84.6%) showed high birefringence alone, indicating a marked decrease in inner scleral fibers with low or mixed birefringence. The remaining 6 eyes (15.4%) had different patterns, including 4 with low birefringence and 2 with a mixture of low and high birefringence. In the optic axis images, 37 of the 39 eyes (94.9%) had vertical fibers and the other 2 eyes (5.1%) had a mixture of vertical and oblique fibers in the sclera surrounding the macula. Streamlined images viewed from outside the eye showed that the fibers in the outer sclera had a vertical orientation (Figure 5D).

### Bidirectional DSMs

Ten eyes of 8 patients with bidirectional DSMs were analyzed (Figure 3 and eFigures 3-7 in Supplement 1). The mean (SD) age of the patients was 59.5 (13.5) years, the mean refractive error of 6 eyes (4 eyes were pseudophakic) was −15.8 (4.5) D, and the mean (SD) AL was 30.8 (1.6) mm. The mean (SD) height of these bidirectional DSMs was 240.6 (100.2) μm, and the mean (SD) width of the DSMs was 3664.6 (982.5) μm. The birefringence images of the sclera of these eyes with bidirectional DSMs varied, but generally the inner scleral fibers were aggregated and thickened with high and low birefringence. In the horizontal and vertical OCT sections, the DSMs had an aggregation of mainly low birefringence fibers in 3 of 10 eyes (30.0%) (Figure 3A and B), an aggregation of mixed high and low birefringent fibers in 4 eyes (40.0%) (eFigure 6 in Supplement 1), an aggregation of high birefringent fibers in 2 eyes (20.0%), and mixed birefringent fibers horizontally and low birefringent fibers vertically in the remaining eye (10.0%).

The vertical scan of the optic axis images at the site of bidirectional DSMs showed that all 10 eyes with bidirectional DSMs had a thickening and protrusion of the horizontal and oblique fibers (Figure 3D and eFigure 6 in Supplement 1). In the horizontal scan images, 7 eyes (70.0%) had an aggregation of horizontal and oblique fibers (Figure 3C and eFigure 6 in Supplement 1), 2 eyes (20.0%) had thickened horizontal fibers alone, and 1 eye (10.0%) had only thickened oblique fibers. Streamlined images viewed from within the eye showed a blend of randomly oriented fibers in the inner sclera at the site of the bidirectional DSMs (eFigures 4 and 7 in Supplement 1). Due to the thickened sclera at the site of a DSM, the outer sclera was only partially visible in 2 eyes (20.0%).

We also examined the scleral findings in areas surrounding the macula. In the vertically scanned images, 9 eyes (90.0%) had vertical fibers with high birefringence in the sclera above and below the DSM, as in myopic eyes without DSM pathology (Figure 3B and D and eFigure 6 in Supplement 1). The other eye (10.0%) had a mixture of horizontal and oblique fibers.

### Horizontal DSMs

Twenty-seven eyes of 25 patients with a horizontal DSM were examined (Figure 4 and eFigures 8 and 9 in Supplement 1). The mean (SD) age of the patients was 63.9 (10.1) years, the mean refractive error of 10 eyes (17 were pseudophakic) was −13.8 (7.2) D, and the mean (SD) AL was 31.2 (1.6) mm. The mean (SD) height of the DSM was 172.2 (73.8) μm, and the mean (SD) width of the DSM was 2980.1 (740.2) μm.

In the eyes with a horizontal DSM that was visible only in the vertical OCT scans, we primarily analyzed the vertical scan images. In these images, the birefringent images revealed an aggregation of mostly low birefringent fibers in 19 of the 27 eyes (70.4%) (Figure 4B) and an aggregation of mixed high and low birefringent fibers in the remaining 8 eyes (29.6%).

The optic axis images of eyes with horizontal DSMs showed an aggregation of fibers with a mixture of horizontal and oblique orientation (Figure 4D) in 22 of the 27 eyes (81.5%). Among the remaining 5 eyes, 4 (14.8%) had an aggregation of horizontal fibers alone and the other eye (3.7%) had an aggregation of oblique fibers alone.

The outer sclera posterior to horizontal DSMs was clearly visible in 18 of 27 eyes (66.7%) in contrast to a limited visibility of the outer sclera posterior to the bidirectional DSMs. In the optic axis images, the outer scleral layer posterior to a DSM had primarily vertically oriented fibers in 12 eyes (66.7%) (Figure 4D), oblique fibers in 5 eyes (27.8%), and a mixture of vertical and oblique fibers in 1 eye (5.6%). None of the 18 eyes had a thickened outer sclera posterior to the DSM, which appeared relatively thin compared with the surrounding areas and appeared compressed by the thickened inner scleral fiber aggregation.

The sclera surrounding the macular area had high birefringence in all 27 eyes with a horizontal DSM. In the optic axis images, 22 eyes (81.5%) had vertically oriented fibers and the remaining 5 eyes (18.5%) had a mixture of vertical and oblique fibers.

## Discussion

The results of this case series showed that PS-OCT images can be used to determine the density and direction of the scleral collagen fibers in highly myopic eyes with a DSM. For control, we first investigated highly myopic eyes without a DSM to examine the inner sclera and eyes with pathologic myopia to observe the outer sclera. Combining the PS-OCT data obtained from these 2 groups, we found that, in general, the inner sclera layer had low-density fibers radiating from the optic nerve. The fibers ran toward and beyond the macula, and the outer sclera consisted of high-density fibers that ran vertically in the posterior segment of the eye.

To our knowledge, this is the first study that determined the orientation and density of the scleral collagen fibers in a wide area of the posterior fundus. Previous studies using PS-OCT in human participants primarily focused on a restricted region around the optic disc^17^ and, in some cases, extended their observations to the macular area^18^ but no further. Willemse et al^17^ examined the architecture of the peripapillary sclera using PS-OCT, and they found radially oriented scleral collagen fibers over a circumferentially arranged layer around the optic disc. Our examination of a broader area of the posterior sclera beyond the macular region found that the radial orientation of the fibers in the inner scleral layer and the intersection of the outer scleral fibers with the inner scleral fibers continued toward the periphery. These findings suggest that the scleral architecture in the posterior fundus may extend from the peripapillary area to the periphery.

The thinning of the inner scleral fiber layer was also noted in eyes with pathologic myopia (Figure 2C and D), although it was not examined in detail. In most eyes with pathologic myopia (30 of 39), almost all of the inner scleral fibers with radial orientation from the optic nerve were not present.

An extremely thin choroid is the main characteristic of eyes with pathologic myopia.^35^ Earlier OCT studies reported that the choroid was almost completely absent, with the remaining large choroidal vessels placed sporadically in eyes with pathologic myopia.^35^ The sclera is relatively avascular; however, the internal sclera receives some nourishment from the choroidal vessels.^1^ Thus, it is likely that the thinning of the inner part of the sclera may result from the extreme thinning of the choroid in eyes with pathologic myopia.

In the PS-OCT images, the sclera at the site of a DSM had an aggregation of scleral fibers mainly with low birefringence or a mixture of low and high birefringence. The optic axis images had a mixture of horizontal and oblique fibers. Although there were some variations among the eyes, these PS-OCT features of aggregated scleral fibers at the DSM were compatible with the findings in the inner scleral layer, as seen in eyes with simple high myopia or with pathologic myopia without a DSM.

An aggregation of fibers in the inner layer of the sclera at the DSM was clearly observed in 66.7% of the eyes with a horizontal DSM. The sclera posterior to the aggregation of inner scleral fibers had high birefringence in the vertical direction. These features were compatible with those seen in the outer sclera of eyes with pathologic myopia without a DSM.

These results suggest that only or mainly the inner scleral fibers aggregated and thickened at the site of a DSM, while the outer sclera was not thickened. Recently, Takahashi et al^39^ reported on an interesting case in which a DSM was split into an inner and outer layer by an intrascleral blood vessel running between the 2 layers. In that eye, OCT showed that only the inner layer of the sclera protruded anteriorly while the outer layer remained in its normal position. The findings in that case supported the findings of the current study.

Why does only the inner sclera thicken in eyes with a DSM? While various suggestions have been proposed regarding the pathogenesis of DSMs, a conclusive explanation has not been determined. Imamura et al^5^ suggested that in eyes with a DSM, the macula tends to remain stationary while other parts of the eye expand during axial elongation in the myopic progression. Fang et al^7^ identified defects in the BM surrounding the DSM, indicating an association between the DSM and these defects. The morphologic characteristics of DSMs together with the BM defects may result from a local relaxation of the posterior sclera so that it is no longer pushed outward by an expanding BM but allowed to protrude slightly inward, leading to the formation of a DSM. In most eyes in our study, the outer sclera was not thickened but was compressed and thinned. It is interesting that these alterations of the inner sclera not only protruded anteriorly but also protruded posteriorly to compress the outer scleral tissue.

To our knowledge, there has not been a study that has examined the different roles of the inner and outer sclera in the development of scleral pathologies. It is possible that the inner sclera with low-density collagen fibers can relax and thicken in the presence of macular BM defects around a DSM. The inner and outer sclera may play different roles in maintaining the scleral curvature and react differently to scleral tensions. The effects of the changes in these tensions should be explored in future research.

Dome-shaped maculopathy has been reported to be associated with various macular complications, such as serous retinal detachments.^3^ Further investigations on the developmental process and progression of DSMs with the use of PS-OCT may provide important clues on the development, treatment, and prevention of DSMs before macular complications occur.

### Limitations

This study has limitations. It was conducted at a single institution (Tokyo Medical and Dental University), possibly introducing selection bias and, thus, limiting the generalizability of the findings. Additionally, the study focused on patients with highly myopic eyes, so the results may not apply to patients with non–highly myopic eyes with a DSM. Despite providing valuable insights, the study’s limited sample sizes, especially in specific subsets, underscore the exploratory nature of findings, emphasizing the necessity for larger, diverse cohorts to validate and extend our observations. Our study, with its cross-sectional design, lacks the ability to capture longitudinal changes, highlighting the need for future research to adopt a longitudinal approach for a more comprehensive understanding of scleral pathologies. Additionally, an age imbalance existed among groups, potentially influencing our findings. Furthermore, the subjective measurements used for primary outcomes lack definitive evidence of reliability or reproducibility. Notably, graders were unmasked to the presence or absence of DSM during assessments, introducing potential bias.

## Conclusions

In this case series, highly myopic eyes with a DSM were made up of thickening and aggregated low-density collagen fibers in the inner layer of the sclera while the outer sclera was not thickened but was compressed. These findings may contribute to new information on the pathogenesis of DSM and other scleral pathologies, such as staphyloma edges.

## Notes

Supplement 1. **eMethods 1.** Details of the PS-OCT Signal Processing **eMethods 2.** Supplemental Explanations of Birefringence and PS-OCT **eTable 1.** Clinical Features of Eyes With and Without Dome-Shaped Macula **eFigure 1.** Images of a Highly Myopic Eye Without Dome-Shaped Macula or Pathologic Myopia (a Supplement to Figure 1) **eFigure 2.** Images of an Eye With Pathologic Myopia and Without DSM (a Supplement to Figure 2) **eFigure 3.** Images of a Highly Myopic Eye With Bidirectional DSM (a Supplement to Figure 3) **eFigure 4.** Streamline Images of the Subject With Bidirectional DSM Shown in Figure 3 and **eFigure 3** **eFigure 5.** Fundus Photograph and PS-OCT Images of a Highly Myopic Eye With Bidirectional DSM **eFigure 6.** Birefringence and Optic Axis Images of the Eye With Bidirectional DSM Shown in **eFigure 5** **eFigure 7.** Streamline Images of the Eye With Bidirectional DSM Shown in **eFigure**s 5 and 6 **eFigure 8.** Fundus Photograph and PS-OCT Images of a Highly Myopic Right Eye With the Horizontal DSM (a Supplement to Figure 4) **eFigure 9.** Streamline Images of the Eye With Horizontal DSM Shown in Figure 4 and **eFigure 8**

Supplement 2.
Data Sharing Statement
