# Supplementary material for: Polarization-Sensitive OCT Imaging of Scleral Abnormalities in Eyes With High Myopia and Dome-Shaped Macula
Source: JAMA Ophthalmol. 2024 Mar 7:e240002. Online ahead of print. doi: 10.1001/jamaophthalmol.2024.0002 (PMC10921350; doi:10.1001/jamaophthalmol.2024.0002)
Supplement: Supplement 1. — eMethods 1. Details of the PS-OCT Signal Processing eMethods 2. Supplemental Explanations of Birefringence and PS-OCT eTable 1. Clinical Features of Eyes With and Without Dome-Shaped Macula eFigure 1. Images of a Highly Myopic Eye Without Dome-Shaped Macula or Pathologic Myopia (a Supplement to Figure 1) eFigure 2. Images of an Eye With Pathologic Myopia and Without DSM (a Supplement to Figure 2) eFigure 3. Images of a Highly Myopic Eye With Bidirectional DSM (a Supplement to Figure 3) eFigure 4. Streamline Images of the Subject With Bidirectional DSM Shown in Figure 3 and eFigure 3 eFigure 5. Fundus Photograph and PS-OCT Images of a Highly Myopic Eye With Bidirectional DSM eFigure 6. Birefringence and Optic Axis Images of the Eye With Bidirectional DSM Shown in eFigure 5 eFigure 7. Streamline Images of the Eye With Bidirectional DSM Shown in eFigures 5 and 6 eFigure 8. Fundus Photograph and PS-OCT Images of a Highly Myopic Right Eye With the Horizontal DSM (a Supplement to Figure 4) eFigure 9. Streamline Images of the Eye With Horizontal DSM Shown in Figure 4 and eFigure 8 [file jamaophthalmol-e240002-s001.pdf]

Supplementary Online Content

Ohno-Matsui K, Igarashi-Yokoi T, Azuma T, et al. Polarization-sensitive optical coherence tomographic imaging of scleral abnormalities in eyes with high myopia and dome-shaped macula. *JAMA Ophthalmol*. 2024;142(2.5):e240002. doi:10.1001/jamaophthalmol.2024.0002

eMethods 1. Details of the PS-OCT Signal Processing

eMethods 2. Supplemental Explanations of Birefringence and PS-OCT

eTable 1. Clinical Features of Eyes With and Without Dome-Shaped Macula

eFigure 1. Images of a Highly Myopic Eye Without Dome-Shaped Macula or Pathologic Myopia (a Supplement to Figure 1)

eFigure 2. Images of an Eye With Pathologic Myopia and Without DSM (a Supplement to Figure 2)

eFigure 3. Images of a Highly Myopic Eye With Bidirectional DSM (a Supplement to Figure 3)

eFigure 4. Streamline Images of the Subject With Bidirectional DSM Shown in Figure 3 and eFigure 3

eFigure 5. Fundus Photograph and PS-OCT Images of a Highly Myopic Eye With Bidirectional DSM

eFigure 6. Birefringence and Optic Axis Images of the Eye With Bidirectional DSM Shown in eFigure 5

eFigure 7. Streamline Images of the Eye With Bidirectional DSM Shown in eFigures 5 and 6

eFigure 8. Fundus Photograph and PS-OCT Images of a Highly Myopic Right Eye With the Horizontal DSM (a Supplement to Figure 4)

eFigure 9. Streamline Images of the Eye With Horizontal DSM Shown in Figure 4 and eFigure 8

This supplementary material has been provided by the authors to give readers additional information about their work.

© 2024 Ohno-Matsui K et al. *JAMA Ophthalmol*.

eMethods 1: Details of the PS-OCT signal processing

The processing flow to calculate the Jones matrix from the measured raw spectra with necessary corrections, including a correction of polarization-mode dispersion, was described previously.[1] The Jones matrix data are preprocessed for denoising by 3-pixel moving average of the Jones matrices along the depth with global phase estimation as the first step of denoising.[2] This Jones matrix could be modeled as Jones matrix of an elliptic retarder, and therefore is not a symmetric matrix in general. Although it does not matter for the calculation of local retardation,[3] it mathematically complicates the derivation of the optic axis. Villiger et al. showed the method to recover the matrix symmetry from the general retarder matrix,[4] thereby effectively converting the elliptic retarder to a linear retarder that the round-trip Jones matrix inherently becomes[5] and thus enabling the approach to resolve axially accumulated Jones matrix into the local Jones matrix.[6,7]

Here, the raster-scanned volumetric data of the Jones matrices are used for the estimation of the correction matrix using Eqs. (8) and (9) of Villiger et al.,[4] and the correction matrix is multiplied to the volumetric data. The corrected volumetric Jones matrix data are processed by Cloude-Pottier decomposition in a moving kernel whose size is 5×15×3 pixels (axial × fast lateral × slow lateral scan directions), and the best estimate of the Jones matrix is extracted from the eigenvector that is paired with the highest eigenvalue of the ensemble-averaged 4×4 covariance matrix for additional denoising of the Jones matrix.[8]

We define a single-trip local Jones matrix $\mathbf{J}_\mathrm{L}$ at the n-th pixel in the axial depth as

$$\mathbf{J}_\mathrm{L}(n) = \mathbf{R}(\theta_n)\mathbf{\Lambda}(\gamma_n)\mathbf{R}(-\theta_n), \tag{1}$$

where $\mathbf{R}(\theta_n)$ is a rotation matrix with an angle of the optic axis θ_n as

$$\mathbf{R}(\theta_n) = \begin{pmatrix} \cos(\theta_n) & -\sin(\theta_n) \\ \sin(\theta_n) & \cos(\theta_n) \end{pmatrix}, \tag{2}$$

$\mathbf{\Lambda}(\gamma_n)$ is a diagonal matrix with the complex value $\gamma_n = \delta_n + i\sigma_n$ that includes phase retardation δ_n and diattenuation σ_n. Using the decreasing phase convention and the symmetric phase convention,[9] $\mathbf{\Lambda}(\gamma_n)$ is described as

© 2024 Ohno-Matsui K et al. *JAMA Ophthalmol.*

$$\mathbf{\Lambda}(\gamma_n) = \begin{pmatrix} e^{-\frac{\gamma_n}{2}} & 0 \\ 0 & e^{+\frac{\gamma_n}{2}} \end{pmatrix}. \quad (3)$$

The parameters in Eq. (1) are what we want to calculate. After the symmetry recovery and denoising of the Jones matrix described in the previous paragraph, the Jones matrix at the *n*-th pixel in the axial depth can be described as (see Eq. (4) of Fan and Yao[7])

$$\begin{aligned}\mathbf{J}_{\mathrm{RT}}(n) &= \mathbf{J}_{\mathrm{ST}}^{\mathrm{T}}(n)\mathbf{J}_{\mathrm{ST}}(n) \\ &= \mathbf{J}_{\mathrm{L}}^{\mathrm{T}}(1)\mathbf{J}_{\mathrm{L}}^{\mathrm{T}}(2)\cdots\mathbf{J}_{\mathrm{L}}^{\mathrm{T}}(n-2)\mathbf{J}_{\mathrm{L}}^{\mathrm{T}}(n-1)\mathbf{J}_{\mathrm{L}}^{\mathrm{T}}(n)\mathbf{J}_{\mathrm{L}}(n)\mathbf{J}_{\mathrm{L}}(n-1)\mathbf{J}_{\mathrm{L}}(n-2)\cdots\mathbf{J}_{\mathrm{L}}(2)\mathbf{J}_{\mathrm{L}}(1) \\ &= \mathbf{R}(\phi_n)\mathbf{\Lambda}(2\rho_n)\mathbf{R}(-\phi_n),\end{aligned} \quad (4)$$

where $\mathbf{J}_{\mathrm{RT}}(n)$ is a round-trip Jones matrix at the depth *n* after the symmetry recovery, $\mathbf{J}_{\mathrm{ST}}(n)$ is a single-trip Jones matrix that includes all effects of the axial depths from 1 to *n*-th pixels, a superscript T indicates the transpose of a matrix, and $\mathbf{R}(\phi_n)$ and $\mathbf{\Lambda}(2\rho_n)$ are obtained by eigendecomposition of $\mathbf{J}_{\mathrm{RT}}(n)$. Since $\mathbf{J}_{\mathrm{RT}}(n)$ is a symmetric matrix, $\mathbf{R}(\phi_n)$ is an orthogonal matrix mathematically. Because of the physical model described above, $\mathbf{R}(\phi_n)$ is a rotation matrix with a rotation angle ϕ_n specifically. $\mathbf{\Lambda}(2\rho_n)$ is a diagonal matrix defined by a complex value $\rho_n = \kappa_n + i\xi_n$ as well as Eq. (3). Since diattenuation is small in most of biological tissues, a modified round-trip Jones matrix at the depth *n*, $\mathbf{J}_{\mathrm{RTM}}(n)$ is described as (see Eq. (6) of Fan and Yao[7])

$$\mathbf{J}_{\mathrm{RTM}}(n) = \mathbf{R}(\phi_n)\mathbf{\Lambda}(2\kappa_n)\mathbf{R}(-\phi_n), \quad (5)$$

where the diattenuation ξ_n is removed from Eq. (4). In the same way, a modified single-trip Jones matrix at the depth *n*, $\mathbf{J}_{\mathrm{STM}}(n)$ is described as

$$\mathbf{J}_{\mathrm{STM}}(n) = \mathbf{R}(\phi_n)\mathbf{\Lambda}(\kappa_n)\mathbf{R}(-\phi_n). \quad (6)$$

Following Eq. (7) of Fan and Yao,[7] the round-trip local Jones matrix $\mathbf{J}_{\mathrm{L}}^{\mathrm{T}}\mathbf{J}_{\mathrm{L}}$ at the *n*-th pixel in the axial depth is described as

$$\mathbf{J}_{\mathrm{L}}^{\mathrm{T}}\mathbf{J}_{\mathrm{L}} = \left[\mathbf{J}_{\mathrm{STM}}^{\mathrm{T}}(n-1)\right]^{-1}\mathbf{J}_{\mathrm{RTM}}(n)[\mathbf{J}_{\mathrm{STM}}(n-1)]^{-1}, \quad (7)$$

© 2024 Ohno-Matsui K et al. *JAMA Ophthalmol.*

where the neighboring pixel at the (n-1)-th depth is used. Here, we assume that the sample has homogeneous birefringent property for Δ pixels along the depth, where Δ is an integer multiple of 2 for convenience. If we use the $\left(n-\frac{\Delta}{2}\right)$-th and $\left(n+\frac{\Delta}{2}\right)$-th depths instead of the (n-1)-th and n-th depths for $\mathbf{J}_{\mathrm{STM}}$ in Eq. (7), a retardation-enhanced Jones matrix $\mathbf{J}_{\mathrm{enhanced}}$ can be defined as

$$\begin{aligned}\mathbf{J}_{\mathrm{enhanced}}(n) &= \left[\mathbf{J}_{\mathrm{STM}}^{\mathrm{T}}\left(n-\frac{\Delta}{2}\right)\right]^{-1} \mathbf{J}_{\mathrm{RTM}}\left(n+\frac{\Delta}{2}\right)\left[\mathbf{J}_{\mathrm{STM}}\left(n-\frac{\Delta}{2}\right)\right]^{-1} \\ &= \left[\mathbf{J}_{\mathrm{STM}}^{\mathrm{T}}\left(n-\frac{\Delta}{2}\right)\right]^{-1} \mathbf{J}_{\mathrm{RTM}\Delta}(n)\left[\mathbf{J}_{\mathrm{STM}}\left(n-\frac{\Delta}{2}\right)\right]^{-1},\end{aligned} \quad (8)$$

where

$$\mathbf{J}_{\mathrm{RTM}\Delta}(n) = \mathbf{R}(\phi_n)\mathbf{\Lambda}(2\Delta\kappa_n)\mathbf{R}(-\phi_n). \quad (9)$$

Since Eq. (8) has higher phase retardation than that of Eq. (7), the phase retardation and optic axis can be estimated robustly, as shown in Makita et al.[3] for the phase retardation measurement previously. By dividing the phase retardation $2\Delta\kappa_n$ derived from Eqs. (8) and (9) by Δ, Eq. (8) is converted to Eq. (7).

To resolve the phase retardation and optic axis for all the depth, the derivation is started from the retinal surface, where the depth is defined as n=1 for each A-scan for convenience. $\mathbf{J}_{\mathrm{RT}}(1)$ in Eq. (4) is estimated from Jones matrices at the retinal surface in each volume using Cloude-Pottier decomposition, and it is rearranged to $\mathbf{J}_{\mathrm{STM}}(1)$ calculated through Eqs. (5) and (6) as

$$\mathbf{J}_{\mathrm{STM}}(1) = \mathbf{R}(\phi_1)\mathbf{\Lambda}(\kappa_1)\mathbf{R}(-\phi_1). \quad (10)$$

Eq. (10) shows a linear retarder that has combined effects of the symmetry recovery and corneal birefringence and can be considered $\mathbf{J}_{\mathrm{L}}(1)$. Using Eqs. (8), (9) and (10) iteratively along the depth, the local retardation δ_n and the optic axis θ_n relative to ϕ_1 can be derived by matrix diagonalization of Eq. (8) in principle. In practice, instead of the matrix diagonalization, Eq. (8) are decomposed using the exponential of the sum of Pauli matrices. This method was demonstrated by Li et al.[10] for the robust estimation of the optic axis in PS-OCT. The general overview of the theory and other applications are described by Chipman et al.[9] Here, we follow the method in Appendix of Li et al.[10] In brief, Eq. (8) is decomposed into retarder and diattenuator vectors with Pauli basis. Since a component of the circular retarder can

© 2024 Ohno-Matsui K et al. *JAMA Ophthalmol.*

be considered noise, it is removed from the retarder vector. A norm of the retarder vector is the phase retardation and is used for the visualization of the local retardation. An angle of the retarder vector that is confined to a linear retarder is calculated on the retarder space and converted to the angle of the optic axis. Since the derived optic axis includes the constant unknown offset ϕ_1 as well as previous demonstrations of optic axis measurement by fiber-based PS-OCT,[11–13] ϕ_1 needs to be determined. We determined ϕ_1 so that the optic axis of the sclera follow the concentric pattern around the optic nerve head.[14] Compared to retinal nerve fiber at the macula that has been used for the reference of the corneal birefringence compensation,[15] the peripapillary sclera was not critically damaged in the eyes of pathologic myopia and could be used for this offset compensation.

The processing algorithm was validated using tissue-like phantoms made of stacked polycarbonate film[16] with rational results as well as previous demonstrations.[4,10,16]

eMethods 2: Supplemental explanations of birefringence and PS-OCT

Because the principles of PS-OCT for birefringence measurement are complicated and most of published documents have been written for optical researchers and engineers, it is hard to get an overview of this technique. In this appendix, supplemental explanations of birefringence and PS-OCT are described to facilitate understanding of the technical background.

Birefringence is an optical property that shows multiple (two or three) refractive indices depending on the incident state of polarized light. Calcite is a representative birefringent crystal, which forms a rhombohedron when the raw crystal is cut along the planes that have weak inter-atomic bonding.[17] Such crystal refracts the light into different directions depending on the polarized components of light and the angle of the crystal. One of the lights is called an ordinary ray, which shows an image through the crystal without lateral shift by the refraction. The other is called an extraordinary ray, which shows the image through the crystal with lateral shift whose orientation rotates with the rotation angle of the crystal. This

phenomenon, which was found by Erasmus Bartholinus in 1669, is attributed to tilted axis of the atomic arrangement to the wavefront of light. The atomic arrangement of the crystal is directly associated with the multiple refractive indices, namely, birefringence. The *optic axis* refers to a direction about which the atoms are arranged symmetrically and is the direction along which plane waves can propagate with a single fixed velocity independent of the state of polarized light, exhibiting only one of the refractive indices of birefringence.[17]

Birefringent crystals are rarely found in biological tissues. Yet birefringence is observed in highly organized fibrous tissues, such as retinal nerve fiber, cornea, and sclera. Birefringence of these fibrous tissues is attributed to a macromolecular optical property called *form birefringence*. Form birefringence arises when there is an ordered arrangement of similar particles or rods of optically isotropic material whose size is large compared with the dimensions of molecules, but small compared with the wavelength of light.[18] Theoretical treatment in respect of electromagnetism was established by Otto Wiener in 1912.[18–20] Because the dimension of each fiber that contributes form birefringence is much smaller than the resolution of OCT in both axial and transversal directions, the measurement of birefringence using OCT in biological tissues enables us finding fibrous tissues in a non-imaging manner even though each fiber is not optically resolved. When polarization-sensitive contrasts that typically include birefringence are extracted from OCT, which is often powered by specially designed optical interferometers for polarization-sensitive measurements, such systems are called polarization-sensitive OCT (PS-OCT).

In a wide sense, birefringence refers to the vectorial nature of the abovementioned optical property including the orientation of the optic axis and the magnitude of the difference in refractive indices. In a narrow sense, however, the magnitude of the birefringence is often called just birefringence as long as it does not cause confusion.

To reiterate, the magnitude of birefringence is represented by a difference of refractive indices along the two optic axes, namely, slow axis and fast axis. The former and the latter exhibit the highest and smallest refractive indices, respectively. Birefringence is always positive when the difference is calculated in this way from the highest to smallest refractive indices. However, it is sometimes calculated irrespective to the order of the refractive indices to be in line with some context,

© 2024 Ohno-Matsui K et al. *JAMA Ophthalmol*.

resulting in both positive and negative values of birefringence. Here, we assume that birefringence is calculated to be always positive. Form birefringence of biological fibrous tissues is typically in the order of 10^{-3}, which is much smaller than birefringence of calcite (~0.172). If the orientation of the fibrous tissues is tilted to the wavefront of light, the same phenomenon where the light is split into two directions as indicated by the ordinary and extraordinary rays occurs in principle. However, the difference of directions between these rays is too small to be detected because of the small magnitude of birefringence, and thus usually safe to be ignored. It is then unnecessary to refer to the terms of the ordinary and extraordinary rays with this simplification, and hereafter our concern is the magnitude of birefringence and the orientation of the optic axis whose component on a plane perpendicular to the light is detectable.

It is known that the slow axis of form birefringence corresponds to the orientation of fibrous tissue.[6,21] It is also known that the higher the density of fibrous tissue, the higher the birefringence.[20,22,23] Because sclera is composed primarily of interwoven collagen fibers,[24–27] the anisotropic orientation of collagen fibers also contributes to measured birefringence.[28]

OCT achieves high axial resolution by using optical interferometry with a broadband light source. In Fourier domain OCT, the interferometric signal is inherently accompanied by signal phase, which is represented as a relative ratio to the center wavelength with a unit of degrees or radians, where one cycle (360 degrees or 2π radians) corresponds to a length of the center wavelength. In other words, the signal phase enables us accessing ultrashort optical path length that is well beyond the axial resolution of a signal intensity. When the signal phases are detected for horizontal and vertical polarizations in PS-OCT, it is possible to measure a phase difference between them and therefore to measure the state of polarization. When the light passes through a birefringent medium, the light experiences different optical path lengths per unit depth depending on the polarization components. PS-OCT can extract the magnitude and the optic axis of birefringence by analyzing how the state of polarization changes along the axial depth. In practice, our PS-OCT illuminates two orthogonally polarized lights to the retina and detects these signals in a parallel manner. Of note, this approach is effective to avoid a degenerated state, where the state of polarization occasionally matches with the optic axis of a birefringent medium and the light cannot experience the influence of the birefringence. The path length difference between the slow and fast axes is called phase retardation or local

© 2024 Ohno-Matsui K et al. *JAMA Ophthalmol.*

(phase) retardation, and is often represented as double-pass phase retardation in degrees per unit depth in PS-OCT,[29] which is also the case of our study. It can be easily converted to the standard unitless representation of birefringence, namely, the difference between refractive indices of the slow and fast axes, by cancelling out the units in the fraction. For example, assuming a center wavelength of 1 μm for simplicity, a double-pass phase retardation of 1 deg/μm is converted as 1 [deg/μm]×1 [μm] / (2×360 [deg]) = 1.4×10^{-3} to be a unitless value, where a factor of 2 in the denominator is to convert double-pass to single-pass representation.

References

1. Yamanari M, Uematsu S, Ishihara K, Ikuno Y. Parallel detection of Jones-matrix elements in polarization-sensitive optical coherence tomography. *Biomed Opt Express*. 2019;10(5):2318-2336.

2. Yamanari M, Tsuda S, Kokubun T, et al. Fiber-based polarization-sensitive OCT for birefringence imaging of the anterior eye segment. *Biomed Opt Express*. 2015;6(2):369-389.

3. Makita S, Yamanari M, Yasuno Y. Generalized Jones matrix optical coherence tomography: performance and local birefringence imaging. *Opt Express*. 2010;18(2):854-876.

4. Villiger M, Braaf B, Lippok N, Otsuka K, Nadkarni SK, Bouma BE. Optic axis mapping with catheter-based polarization-sensitive optical coherence tomography. *Optica*. 2018;5(10):1329-1337.

5. Jiao S, Yu W, Stoica G, Wang LV. Contrast Mechanisms in Polarization-Sensitive Mueller-Matrix Optical Coherence Tomography and Application in Burn Imaging. *Appl Opt*. 2003;42(25):5191-5197.

6. Todorović M, Jiao S, Wang LV, Stoica G. Determination of local polarization properties of biological samples in the presence of diattenuation by use of Mueller optical coherence tomography. *Opt Lett*. 2004;29(20):2402-2404.

7. Fan C, Yao G. Imaging myocardial fiber orientation using polarization sensitive optical coherence tomography. *Biomed Opt Express*. 2013;4(3):460-465.

© 2024 Ohno-Matsui K et al. *JAMA Ophthalmol*.

8. Yamanari M, Tsuda S, Kokubun T, et al. Estimation of Jones matrix, birefringence and entropy using Cloude-Pottier decomposition in polarization-sensitive optical coherence tomography. *Biomed Opt Express*. 2016;7(9):3551-3573.

9. Chipman RA, Lam WST, Young G. *Polarized Light and Optical Systems*. CRC Press; 2018.

10. Li Q, Karnowski K, Noble PB, et al. Robust reconstruction of local optic axis orientation with fiber-based polarization-sensitive optical coherence tomography. *Biomed Opt Express*. 2018;9(11):5437-5455.

11. Lu Z, Matcher SJ. Absolute fast axis determination using non-polarization-maintaining fiber-based polarization-sensitive optical coherence tomography. *Opt Lett*. 2012;37(11):1931-1933.

12. Park BH, Pierce MC, Cense B, de Boer JF. Optic axis determination accuracy for fiber-based polarization-sensitive optical coherence tomography. *Opt Lett*. 2005;30(19):2587-2589.

13. Adams DC, Hariri LP, Miller AJ, et al. Birefringence microscopy platform for assessing airway smooth muscle structure and function in vivo. *Sci Transl Med*. 2016;8(359):359ra131.

14. Willemse J, Grafe MGO, Verbraak FD, de Boer JF. In Vivo 3D Determination of Peripapillary Scleral and Retinal Layer Architecture Using Polarization-Sensitive Optical Coherence Tomography. *Transl Vis Sci Technol*. 2020;9(11):21-21.

15. Pircher M, Gotzinger E, Baumann B, Hitzenberger CK. Corneal birefringence compensation for polarization sensitive optical coherence tomography of the human retina. *J Biomed Opt*. 2007;12(4):041210.

16. Liu X, Beaudette K, Wang X, Liu L, Bouma BE, Villiger M. Tissue-like phantoms for quantitative birefringence imaging. *Biomed Opt Express*. 2017;8(10):4454-4465.

17. Hecht E. *Optics*. Pearson Education; 2017.

© 2024 Ohno-Matsui K et al. *JAMA Ophthalmol*.

18. Born M, Wolf E. *Principles of Optics: Electromagnetic Theory of Propagation, Interference and Diffraction of Light (7th Edition)*. Cambridge University Press; 1999.

19. Oldenbourg R, Ruiz T. Birefringence of macromolecules. Wiener's theory revisited, with applications to DNA and tobacco mosaic virus. *Biophys J*. 1989;56(1):195-205.

20. Oldenbourg R, Salmon ED, Tran PT. Birefringence of Single and Bundled Microtubules. *Biophys J*. 1998;74(1):645-654.

21. Kemp NJ, Park J, Zaatari HN, Rylander HG, Milner TE. High-sensitivity determination of birefringence in turbid media with enhanced polarization-sensitive optical coherence tomography. *J Opt Soc Am A*. 2005;22(3):552-560.

22. Zhou Q, Knighton RW. Light scattering and form birefringence of parallel cylindrical arrays that represent cellular organelles of the retinal nerve fiber layer. *Appl Opt*. 1997;36(10):2273-2285.

23. Huang XR, Knighton RW. Microtubules Contribute to the Birefringence of the Retinal Nerve Fiber Layer. *Invest Ophthalmol Vis Sci*. 2005;46(12):4588-4593.

24. Komai Y, Ushiki T. The three-dimensional organization of collagen fibrils in the human cornea and sclera. *Invest Ophthalmol Vis Sci*. 1991;32(8):2244-2258.

25. Meek KM. The Cornea and Sclera. In: Fratzl P, ed. *Collagen*. ; 2008:359-396.

26. Summers Rada JA, Shelton S, Norton TT. The sclera and myopia. *Exp Eye Res*. 2006;82(2):185-200.

27. Jan NJ, Lathrop K, Sigal IA. Collagen Architecture of the Posterior Pole: High-Resolution Wide Field of View Visualization and Analysis Using Polarized Light Microscopy. *Invest Ophthalmol Vis Sci*. 2017;58(2):735-744.

28. Li Q, Karnowski K, Untracht G, et al. Vectorial birefringence imaging by optical coherence microscopy for assessing fibrillar microstructures in the cornea and limbus. *Biomed Opt Express*. 2020;11(2):1122-1138.

29. Cense B, Chen TC, Park BH, Pierce MC, de Boer JF. Thickness and Birefringence of Healthy Retinal Nerve Fiber Layer Tissue Measured with

© 2024 Ohno-Matsui K et al. *JAMA Ophthalmol*.

Polarization-Sensitive Optical Coherence Tomography. *Invest Ophthalmol Vis Sci*. 2004;45(8):2606-2612.

© 2024 Ohno-Matsui K et al. *JAMA Ophthalmol*.

eTable 1: Clinical features of eyes with and without dome-shaped macula

	Simple high myopia	Pathologic myopia		
		Without DSM	Horizontal DSM	Bidirectional DSM
Number of eyes (patients)	13 (8)	39 (31)	27 (25)	10 (8)
Age (years-old, mean ± SD)	51.6 ± 16.0 (26-82)	63.7 ± 11.2 (41-94)	63.9 ± 10.4 (48-85)	59.5 ± 13.5 (39-78)
Axial length (mm, mean ± SD)	28.5 ± 1.2 (26.8-31.2)	30.4 ± 1.5 (27.9-35.6)	31.2 ± 1.7 (27.99-34.56)	30.8 ± 1.6 (28.7-33.4)
Category of myopic maculopathy*				
C1 (tessellated fundus)	13	0	0	0
C2 (diffuse atrophy)	0	27	9	7
C3 (patchy atrophy)	0	9	12	3
C4 (macular atrophy)	0	3	6	0
Complications of DSM				
Serous RD	0	0	0	2
MNV	0	15	8	0
Foveal RS	0	6	2	0
Extrafoveal RS	0	8	3	3
Macular Hole	0	2	0	0

*; according to META-PM study classification.

DSM; dome-shaped macula, RD; retinal detachment, MNV; macular neovascularization, RS; retinoschisis, SD; standard deviation.

© 2024 Ohno-Matsui K et al. *JAMA Ophthalmol*.

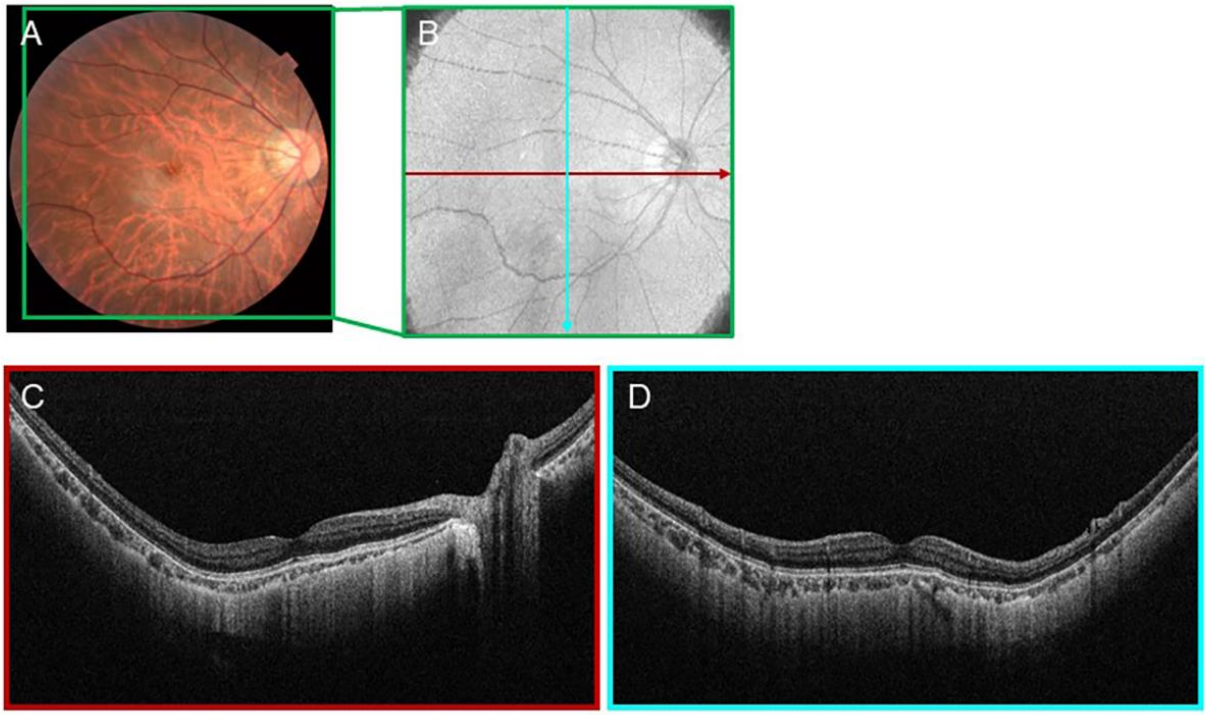

eFigure 1. Images of a highly myopic eye without dome-shaped macula or pathologic myopia (a supplement to Figure 1).

A. Right fundus of the 30-year-old man with a refractive error of -14.9 diopters and an axial length of 28.9 mm shows tessellated fundus alone without myopic atrophic lesions. **B.** Arrows on the *en face* OCT intensity projection show the location of PS-OCT scans that include the fovea. **C and D.** Horizontal (C) as well as vertical (D) OCT sections across the fovea visualize the inner part of the sclera while the scleral posterior surface is not visible. Here, (C) and (D) are measured separately with horizontal and vertical raster scans, respectively, and thus (D) has high-quality dense A-scans.

© 2024 Ohno-Matsui K et al. *JAMA Ophthalmol*.

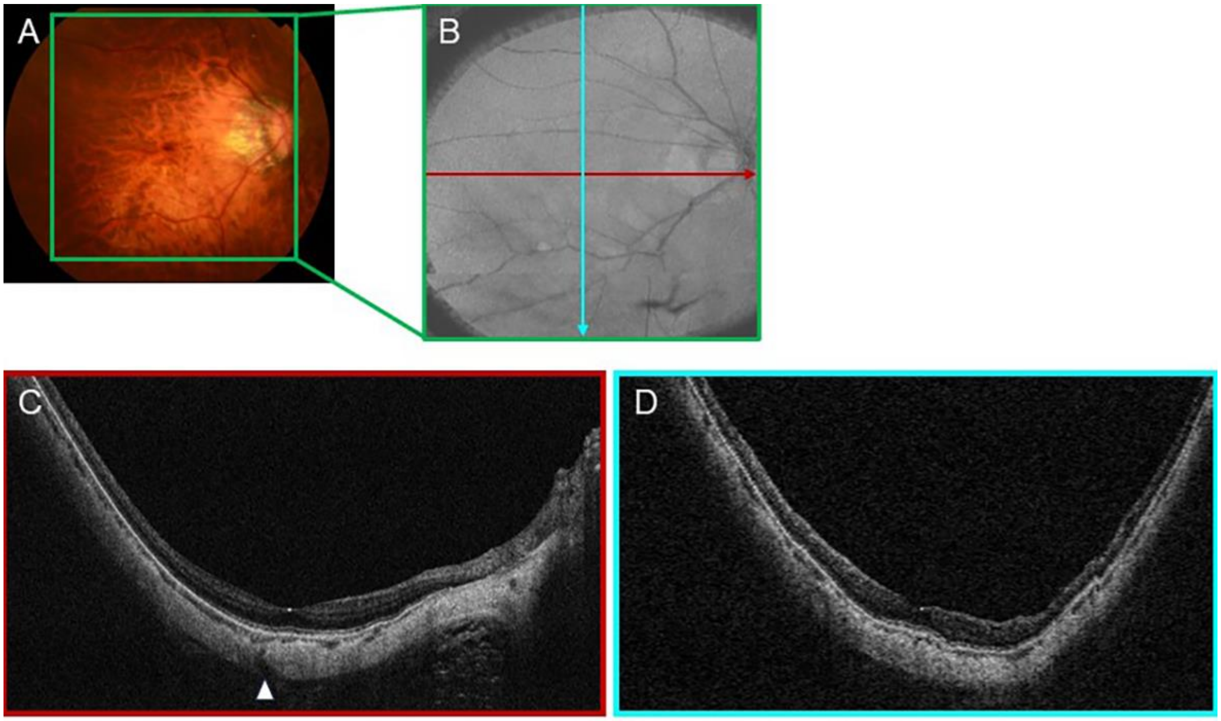

eFigure 2. Images of an eye with pathologic myopia and without DSM (a supplement to Figure 2).

The labeling of the images A through D are same as in eFigure 1. In (A), Right fundus of a 46-year-old woman with a refractive error of -23.5 diopters and an axial length of 32.0 mm shows diffuse choroidal atrophy. Horizontal (C) as well as vertical (D) OCT sections across the fovea show that the sclera is generally thin, and it is visible in its entire thickness. The blood vessel emissary, indicated by a white arrowhead in (C), penetrates the sclera near the fovea. (D) was created by re-slicing the horizontal raster-scanned volume, and thus they have a lower A-scan density.

© 2024 Ohno-Matsui K et al. *JAMA Ophthalmol*.

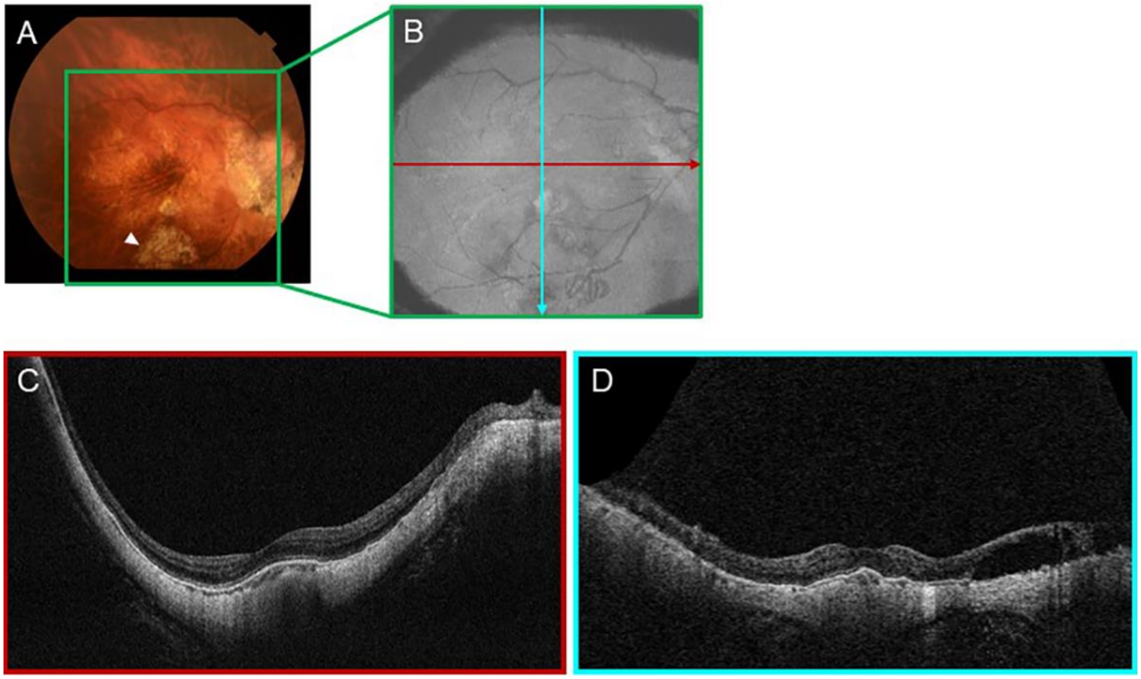

eFigure 3. Images of a highly myopic eye with bidirectional DSM (a supplement to Figure 3).

The labeling of (A)-(D) are same as in eFigure 1. In (A), right fundus of a 76-year-old woman with an intraocular lens and an axial length of 30.1 mm shows diffuse choroidal atrophy. An area of patchy choroidal atrophy can be seen inferior to the macula, indicated by a white arrowhead. Horizontal (C) as well as vertical (D) OCT sections across the fovea show the inward bulge indicating bidirectional DSM. The outer surface of the sclera is visible except in the area of the DSM. In the DSM area, the outer surface of sclera is not clearly seen due to the thickening by the DSM. The DSM height is 233 μm. Note that the vertically resliced section in (D) is motion corrected numerically, which flattened the retinal image as a side effect.

© 2024 Ohno-Matsui K et al. *JAMA Ophthalmol*.

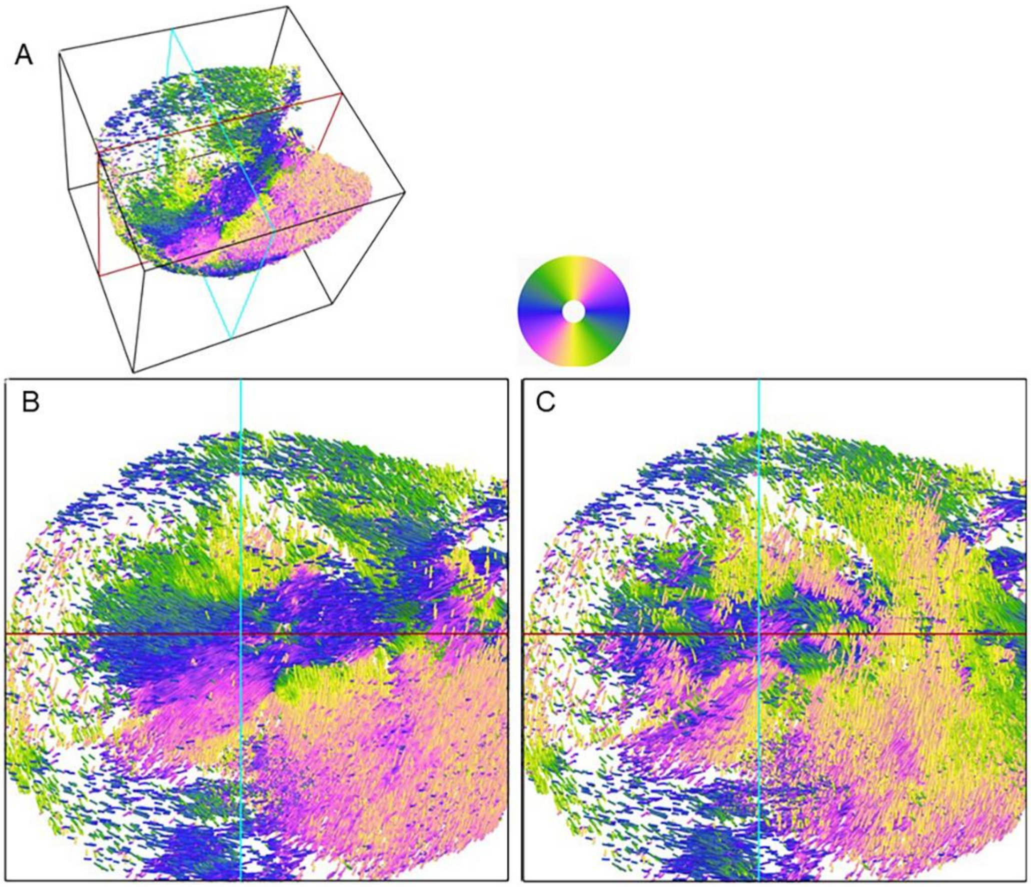

eFigure 4. Streamline images of the subject with bidirectional DSM shown in Figure 3 and eFigure 3.

Streamline images recorded with an oblique angle of the camera (A), from the interior of the eye (B), and from the exterior of the eye (C) rendered by ParaView. The sclera posterior to the DSM is not fully visible in (C), and this image mainly shows the DSM viewed from the outside. Vertically running fibers (yellow) in the outer sclera are seen only between the optic nerve and the macula. In (B) and (C), the DSM shows a mixture of horizontal and oblique fibers.

© 2024 Ohno-Matsui K et al. *JAMA Ophthalmol*.

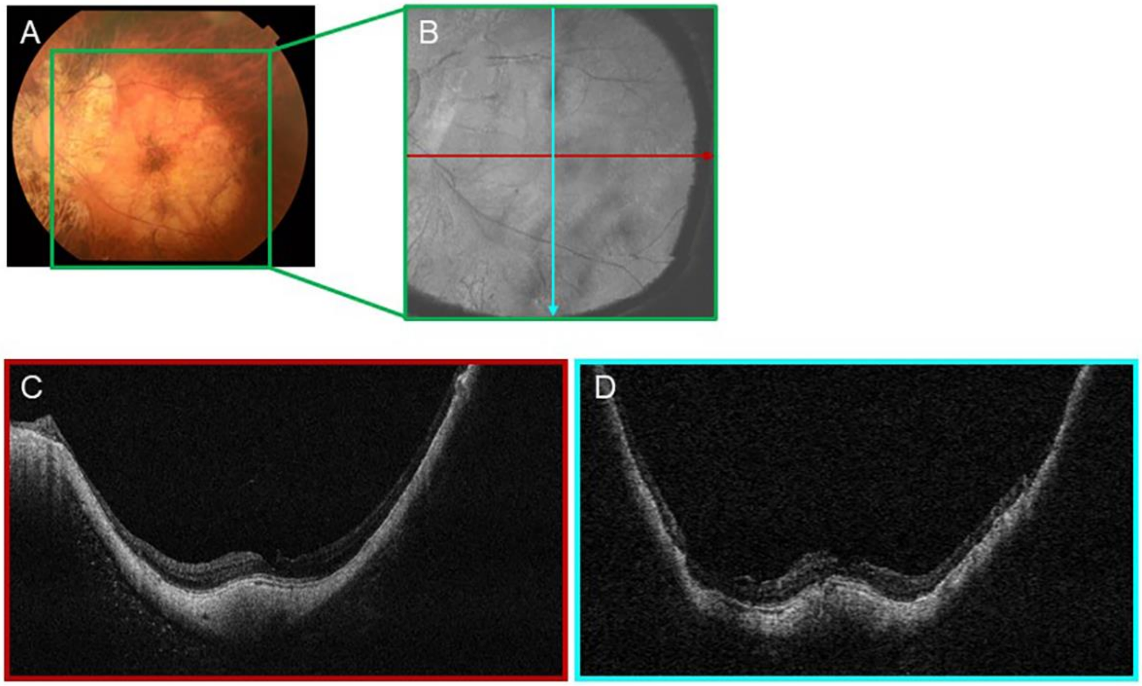

eFigure 5. Fundus photograph and PS-OCT images of a highly myopic eye with bidirectional DSM.

Labels A to D are the same as in eFigure 1. The subject was a 76-year-old woman with an intraocular lens and an axial length of 32.3 mm showing diffuse choroidal atrophy. Horizontal (C) and vertical (D) OCT images across the fovea showing an inward bulge suggesting bidirectional DSM. The outer surface of the sclera is visible except for the DSM area where the sclera is thick. The DSM height is 176 µm.

© 2024 Ohno-Matsui K et al. *JAMA Ophthalmol*.

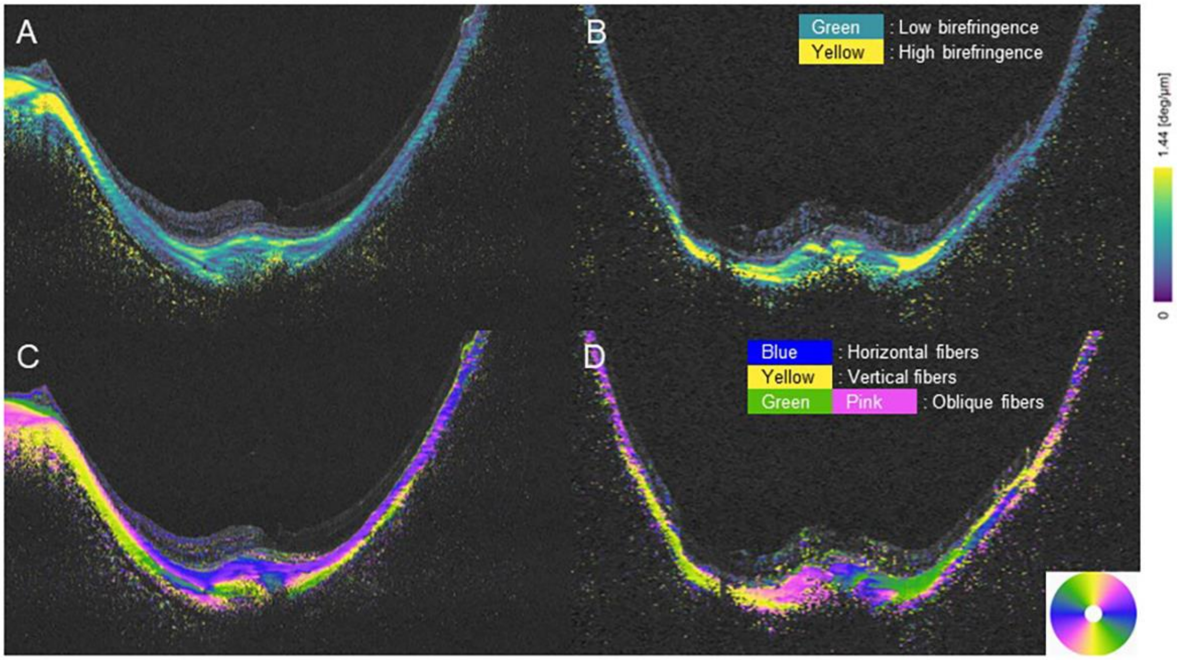

eFigure 6. Birefringence and optic axis images of the eye with bidirectional DSM shown in eFigure 5.

The horizontal section of the birefringence image across the fovea (A) shows low birefringent fibers (green) in the DSM, and high birefringent fibers (yellow) are sporadically seen. Highly birefringent fibers are also seen temporal to the optic nerve. The vertical section (B) across the fovea shows a mixture of low (green) and high (yellow) birefringent fibers at the DSM. In contrast, fibers with high birefringence (yellow) are seen superior and inferior to the DSM. In the horizontal scanned image of the optic axis image (C), the DSM shows a mixture of horizontal (blue) and oblique fibers (pink). These fibers in the inner sclera are widely seen nasal and temporal to the DSM along the inner sclera. Vertical fibers (yellow) are seen in the outer sclera posterior to the DSM. In a vertical scanned image (D), a mixture of horizontal (blue) and oblique fibers (green and pink) is seen at the DSM. The sclera superior and inferior to the DSM consists mainly of vertical fibers (yellow) in the outer sclera.

© 2024 Ohno-Matsui K et al. *JAMA Ophthalmol*.

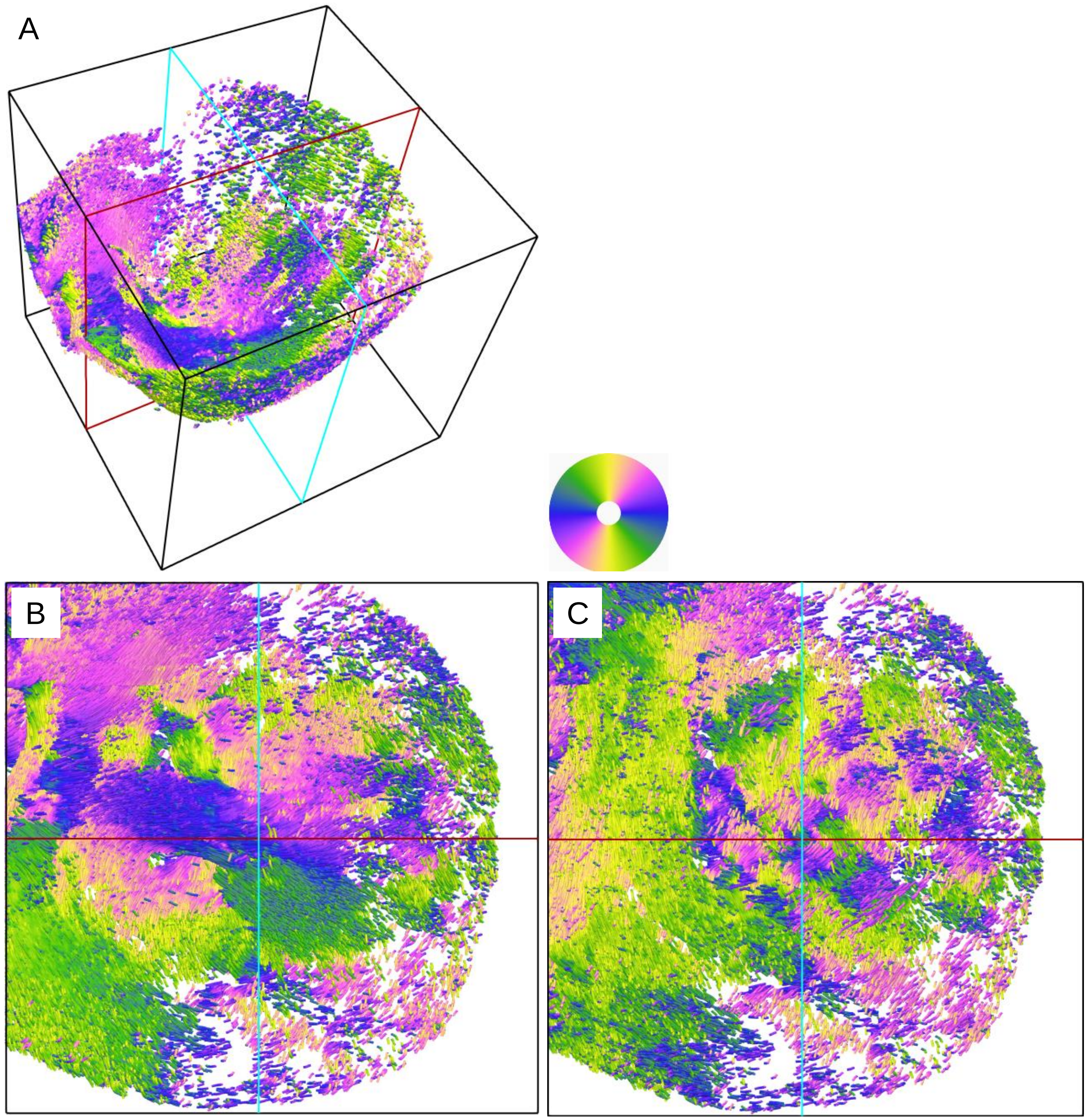

eFigure 7. Streamline images of the eye with bidirectional DSM shown in eFigures 5 and 6.

Streamline images recorded with an oblique angle of the camera (A), from the interior of the eye (B), and from the exterior of the eye (C) rendered by ParaView. The sclera posterior to the DSM is not fully visible, and thus only the DSM can be seen from the outside (C). Vertically running fibers (yellow) in the outer sclera are

© 2024 Ohno-Matsui K et al. *JAMA Ophthalmol*.

seen only between the optic nerve and the macula in image C. In images B and C, the DSM has a mixture of horizontal and oblique fibers.

© 2024 Ohno-Matsui K et al. *JAMA Ophthalmol*.

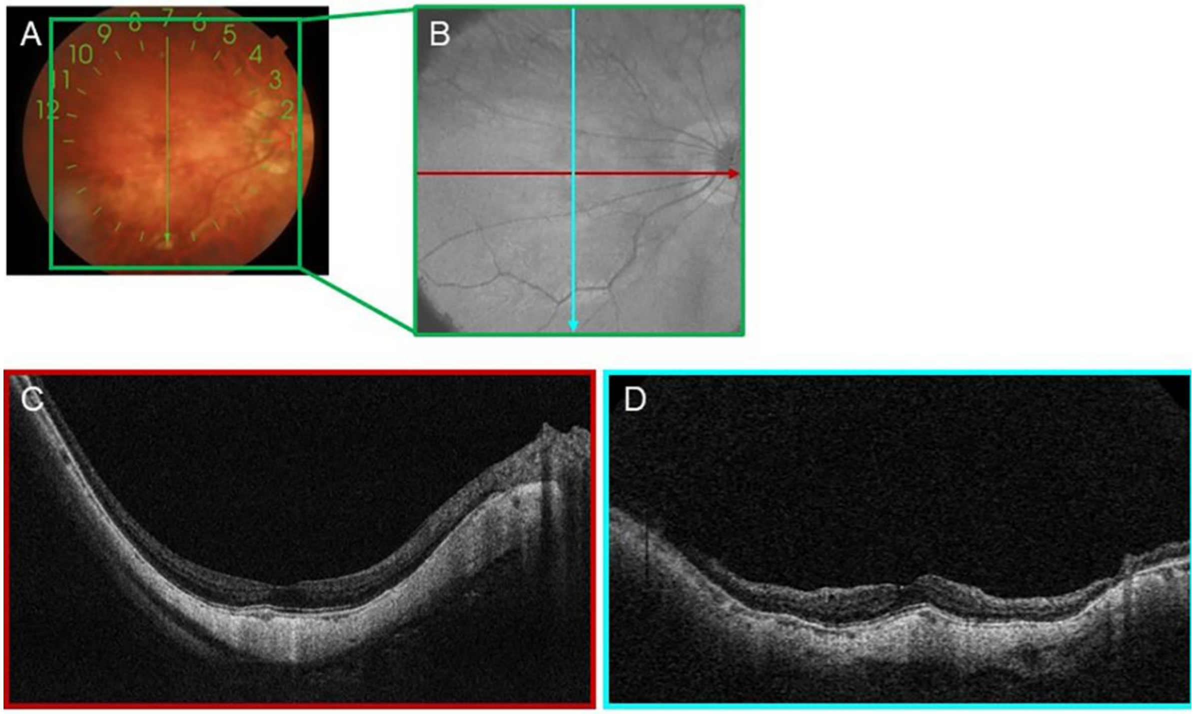

eFigure 8. Fundus photograph and PS-OCT images of a highly myopic right eye with the horizontal DSM (a supplement to Figure 4).

Labels A through D are the same as in eFigure 1. Horizontal (C) and vertical (D) OCT images across the fovea showing that the inward bulge is seen only in the vertical section indicating a horizontal DSM. The outer surface of the sclera is clearly seen in all areas. The maximum DSM height is 68 µm. Note that the vertically resliced section in (D) is motion corrected numerically which flattened the retinal image as a side effect.

© 2024 Ohno-Matsui K et al. *JAMA Ophthalmol*.

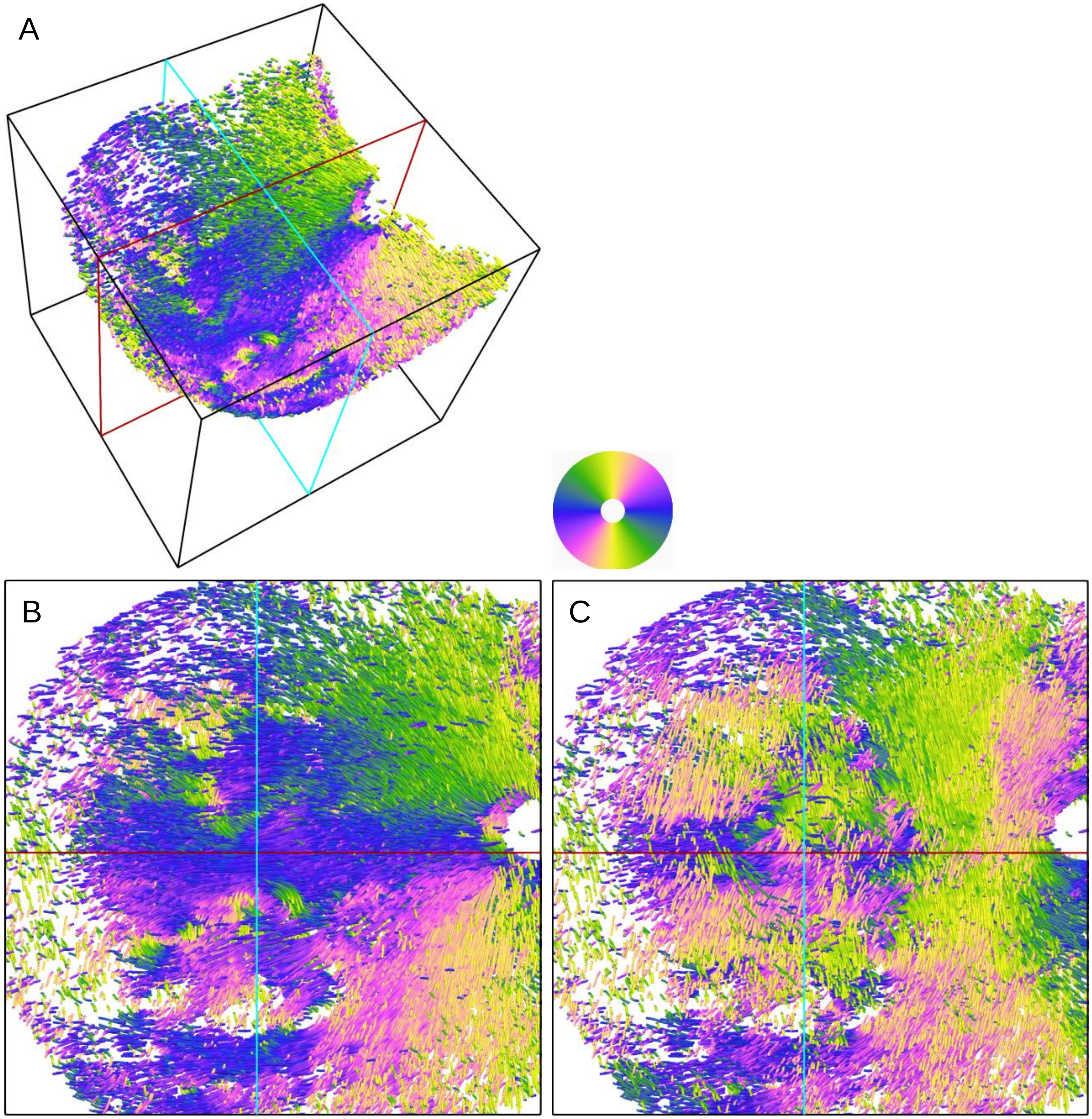

eFigure 9. Streamline images of the eye with horizontal DSM shown in Figure 4 and eFigure 8.

Streamline images recorded with an oblique angle of the camera (A), from the interior of the eye (B), and from the exterior of the eye (C) rendered by ParaView.

© 2024 Ohno-Matsui K et al. *JAMA Ophthalmol*.
