# Supplementary material for: Polarization-Sensitive OCT Imaging of Scleral Abnormalities in Eyes With High Myopia and Dome-Shaped Macula
Source: JAMA Ophthalmol. 2024 Mar 7:e240002. Online ahead of print. doi: 10.1001/jamaophthalmol.2024.0002 (PMC10921350; doi:10.1001/jamaophthalmol.2024.0002)
Supplement: Supplement 2. — Data Sharing Statement [file jamaophthalmol-e240002-s002.pdf]

Data Sharing Statement

Ohno-Matsui. Polarization-Sensitive Optical Coherence Tomographic Imaging of Scleral Abnormalities in Eyes With High Myopia and Dome-Shaped Macula. *JAMA Ophthalmol*. Published March 07, 2024. doi:10.1001/jamaophthalmol.2024.0002

Data
Data available: No
